# Low-dose ethanol increases aflatoxin production due to the *adh1*-dependent incorporation of ethanol into aflatoxin biosynthesis

**DOI:** 10.1016/j.isci.2023.106051

**Published:** 2023-01-23

**Authors:** Tomohiro Furukawa, Masayo Kushiro, Hiroyuki Nakagawa, Hirofumi Enomoto, Shohei Sakuda

**Affiliations:** 1 Institute of Food Research, National Agriculture and Food Research Organization (NARO), 2-1-12 Kannon-dai, Tsukuba-shi, Ibaraki 305-8642, Japan; 2 Research Center for Advanced Analysis, NARO, 2-1-12 Kannon-dai, Tsukuba-shi, Ibaraki 305-8642, Japan; 3 Department of Biosciences, Faculty of Science and Engineering, Teikyo University, 1-1 Toyosatodai, Utsunomiya-shi, Tochigi 320-8551, Japan; 4 Advanced Instrumental Analysis Center, Teikyo University, 1-1 Toyosatodai, Utsunomiya-shi, Tochigi 320-8551, Japan

**Keywords:** Microbial metabolism, Mycology

## Abstract

Aflatoxins are toxic secondary metabolites produced by some aspergilli, including *Aspergillus flavus*. Recently, ethanol has attracted attention as an agent for the control of aflatoxin contamination. However, as aflatoxin biosynthesis utilizes acetyl coenzyme A, ethanol may be conversely exploited for aflatoxin production. Here, we demonstrated that not only the ^13^C of labeled ethanol, but also that of labeled 2-propanol, was incorporated into aflatoxin B_1_ and B_2_, and that ethanol and 2-propanol upregulated aflatoxin production at low concentrations (<1% and <0.6%, respectively). In the alcohol dehydrogenase gene *adh1* deletion mutant, the ^13^C incorporation of labeled ethanol, but not labeled 2-propanol, into aflatoxin B_1_ and B_2_ was attenuated, indicating that the alcohols have different utilization pathways. Our results show that *A. flavus* utilizes ethanol and 2-propanol as carbon sources for aflatoxin biosynthesis and that *adh1* indirectly controls aflatoxin production by balancing ethanol production and catabolism.

## Highlights

• Ethanol and 2-propanol increase aflatoxin production at low concentrations
• Ethanol and 2-propanol are incorporated into aflatoxin biosynthesis via acetyl-CoA
• Increments in aflatoxin production by ethanol are equal to ethanol-derived aflatoxin
• *adh1* plays a major role in the utilization of ethanol, but not 2-propanol

## Graphical abstract

**Figure:**
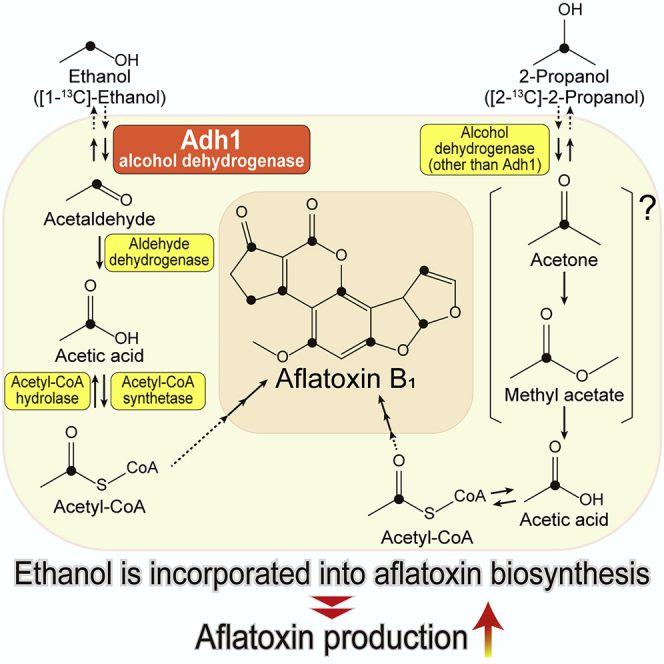

## Introduction

Aflatoxins are highly toxic fungal secondary metabolites produced by some *Aspergillus* species, including *Aspergillus flavus* and *Aspergillus parasiticus*. These aflatoxigenic fungi infect and contaminate crops such as maize, peanut, cotton, and tree nuts.^1^ In addition to posing high health risks for humans and livestock, aflatoxin contamination results in significant economic losses because of crop disposal.^2^ Thus, effective methods for its control are required. For this purpose, elucidation of the regulatory mechanism underlying aflatoxin production is important.

Aflatoxin is biosynthesized in at least 18 enzyme steps using 10 molecules of acetyl coenzyme A (acetyl-CoA) and 2 molecules of *S*-adenosylmethionine; 25 or more genes clustered in a 70-kb region on one chromosome are responsible for this process.^3^^,^^4^ Acetyl-CoA is produced through the oxidative decarboxylation of pyruvate via acetaldehyde and acetate, the β-oxidation of fatty acids in mitochondria and peroxisomes, and conversion from citrate.^5^^,^^6^ *A. flavus* produces mainly aflatoxin B_1_, the most potent naturally formed carcinogen, and a lesser amount of aflatoxin B_2_, a dihydro derivative of aflatoxin B_1_.^7^ Ethanol, the most familiar form of alcohol, is recognized as an inhibitor of microorganism growth and viability. High levels of ethanol increase the permeability of plasma membranes, dissipate ionic gradients across these membranes, and eliminate their electrochemical potential.^8^^,^^9^ These effects halt nutrient and waste exchange with the environment, leading to growth inhibition and cell death. Recently, 3.5% ethanol was reported to reduce the fungal biomass, upregulate the expression of genes related to the oxidative stress response, and downregulate the expression of aflatoxin biosynthetic cluster genes, inhibiting aflatoxin B_1_ production, in *A. flavus* strain NRRL 3357.^10^ These researchers found that 3–4% ethanol reduced aflatoxin B_1_ production and proposed that ethanol is applicable for the control of aflatoxin contamination.^10^

However, ethanol is also a product of alcoholic fermentation. *A. flavus* has enzymes involved in this fermentation pathway: pyruvate decarboxylase, which catalyzes the decarboxylation of pyruvate to acetaldehyde, and alcohol dehydrogenase, which facilitates the interconversion of acetaldehyde to ethanol.^11^^,^^12^ As *A. flavus* has genes encoding aldehyde dehydrogenase, which catalyzes the irreversible oxidation of acetaldehyde to acetate,^13^ it is reasonable to consider that ethanol is oxidized to acetate, which is then utilized in aflatoxin biosynthesis. Consistent with this inference, evidence suggests that ethanol is associated positively with aflatoxin production. Gupta et al.^14^ reported that ethanol inhibited the incorporation of [1-^14^C]-acetate into aflatoxin biosynthesis, likely because of the formation of acetyl-CoA from ethanol, which diluted the [1-^14^C]-acetate label. Bennett et al.^15^ reported that 10 and 100 mM ethanol increased *A. parasiticus* aflatoxin production in the presence of glucose.

A considerable amount of research on the incorporation of labeled acetate into aflatoxin biosynthesis has been conducted, revealing that maxima of nine and seven aflatoxin B_1_ carbon atoms are ^13^C labeled by [1-^13^C]- and [2-^13^C]-acetate, respectively.^16^^,^^17^^,^^18^^,^^19^ In this study, we used ^13^C-labeled ethanol to investigate the availability of ethanol for aflatoxin biosynthesis.

## Results

### Low-dose ethanol and 2-propanol increased aflatoxin production

**Figure 1:**
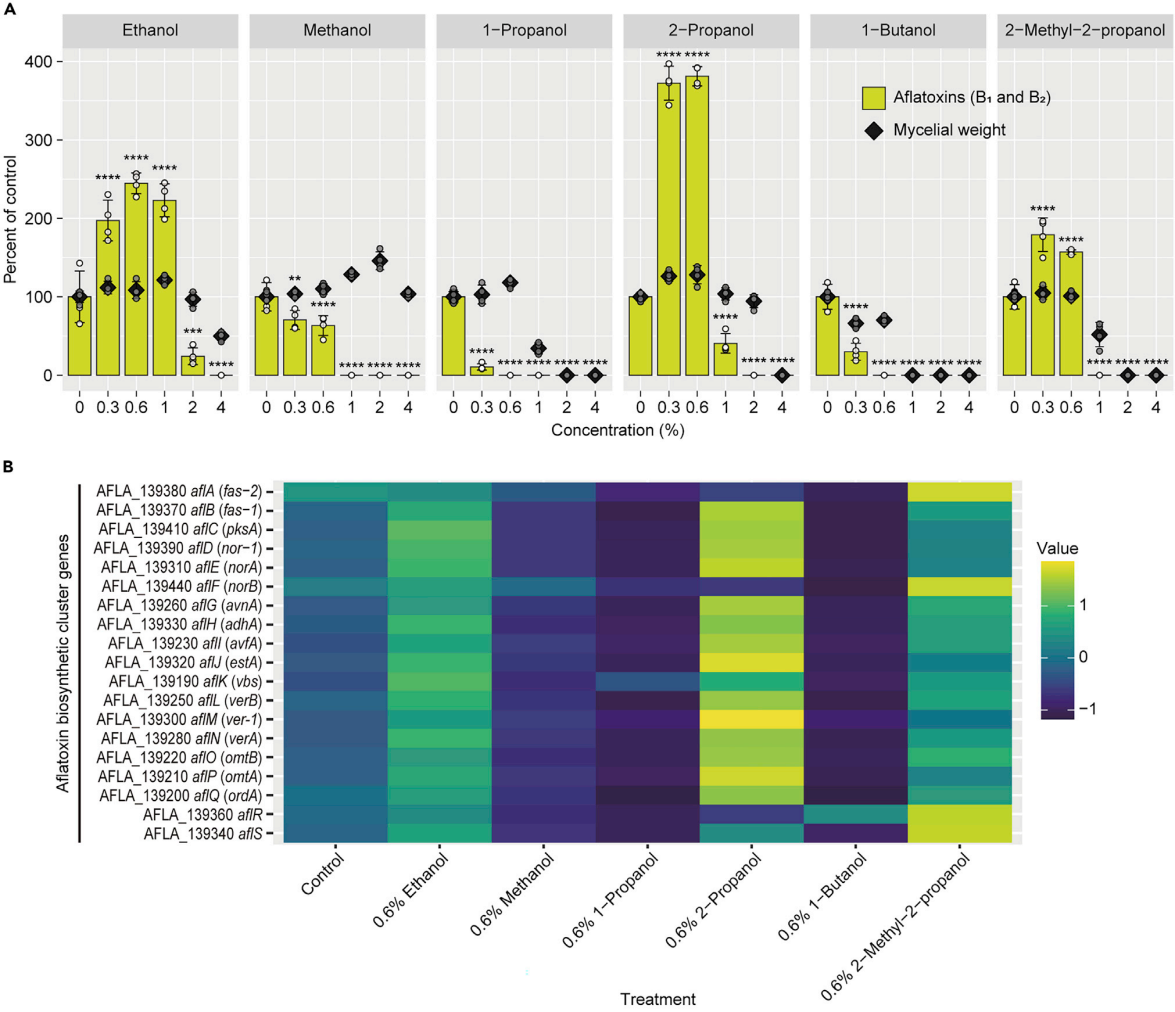
Effects of low-molecular-weight alcohols on the aflatoxin production and gene expression of aflatoxin biosynthetic cluster genes of *Aspergillus flavus* IFM 47798 (A) Effects of alcohols on aflatoxin B_1_ and B_2_ production and mycelial dry weight. Mean ± SD, n = 4. ∗∗p< 0.01, ∗∗∗p< 0.001, ∗∗∗∗p< 0.0001 versus control, ANOVA followed by Dunnett’s test. (B) Heatmap of relative expression patterns of aflatoxin biosynthetic cluster genes with the addition of 0.6% of each alcohol, determined by RT-qPCR. mRNA levels were standardized, and averages of n = 3 are shown. See also Figure S1.

To examine the effect of low-molecular-weight alcohols on aflatoxin production, *A. flavus* strain IFM 47798 was cultured with the addition of 0–4% ethanol, methanol, 1-propanol, 2-propanol, 1-butanol, or 2- methyl-2-propanol for 48 h, and the aflatoxin B_1_ and B_2_ in culture supernatants and mycelial dry weights were measured (Figure 1A). Methanol, 1-propanol, and 1-butanol significantly inhibited aflatoxin production at all concentrations tested. High concentrations of ethanol, 2-propanol, and 2-methyl-2-propanol reduced aflatoxin B_1_ and B_2_ production, whereas low concentrations of these alcohols (0.3–1.0% for ethanol, 0.3–0.6% for 2-propanol, and 0.3–0.6% for 2-methyl-2-propanol) significantly increased it. At high concentrations, all alcohols except methanol reduced the mycelial weight.

Although to a lesser degree than in *A. flavus* IFM 47798, aflatoxin production in *A. parasiticus* NRRL 2999 was also significantly increased with 0.3% ethanol and 0.3–0.6% 2-propanol (Figure S1). 2-Methyl-2-propanol, methanol, 1-propanol, and 1-butanol showed concentration-dependent aflatoxin production inhibitory activity in this strain.

To investigate the effects of these alcohols on aflatoxin biosynthetic cluster gene expression, fungal mRNA was collected from *A. flavus* cultured with 0.6% of each alcohol*,* and the mRNA levels were determined by quantitative reverse-transcription polymerase chain reaction (RT-qPCR; Figure 1B). Ethanol, 2-propanol, and 2-methyl-2-propanol tended to increase the mRNA levels of all genes examined, whereas methanol, 1-propanol, and 1-butanol tended to decrease them, consistent with the effects of these alcohols on aflatoxin production.

### ^13^C of labeled ethanol was incorporated into aflatoxin B_1_

**Figure 2:**
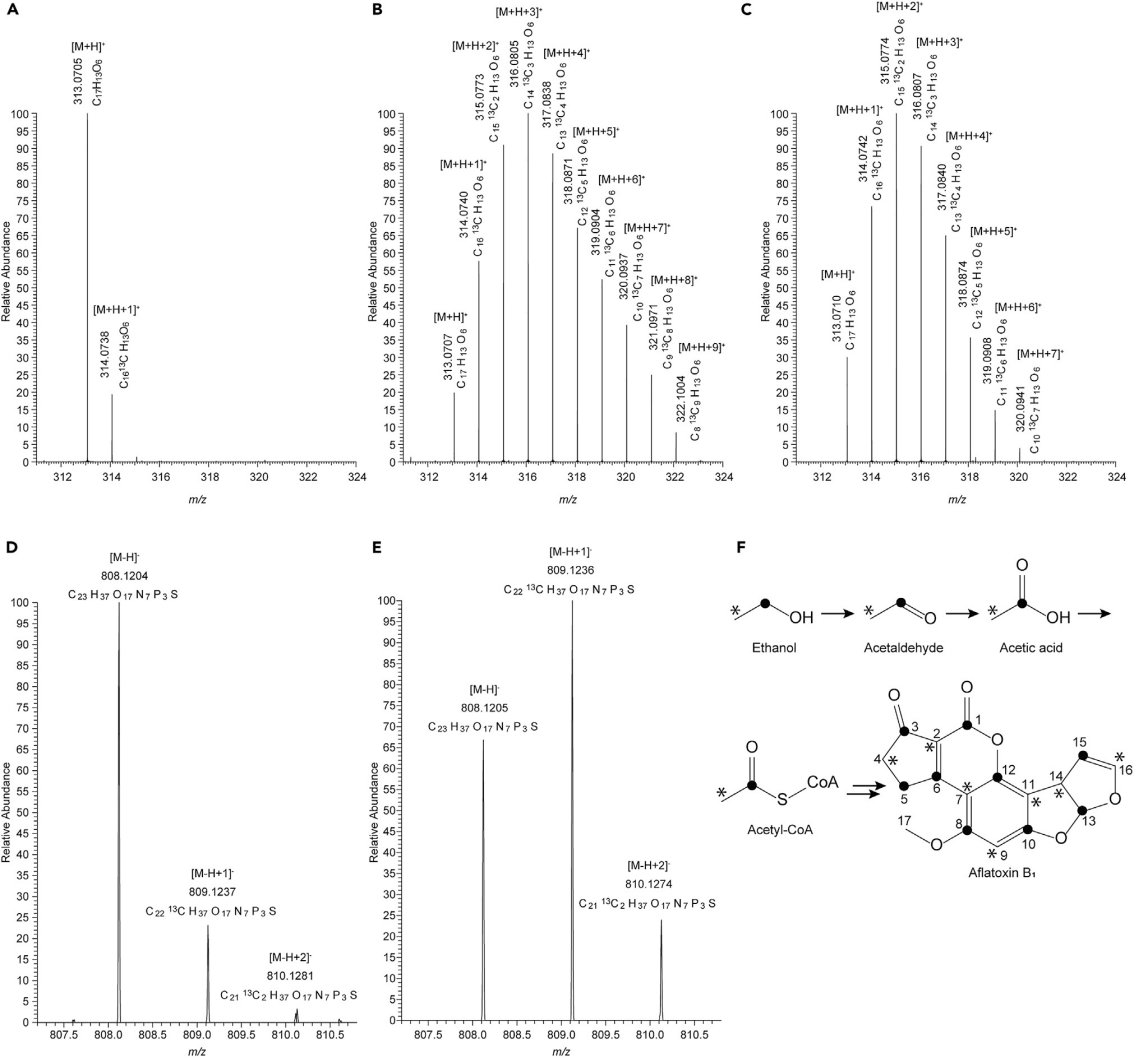
[1-^13^C]-ethanol was incorporated into aflatoxin B_1_ biosynthesis via acetyl-CoA (A–C) Mass spectra of aflatoxin B_1_ extracted from control culture and cultures supplemented with [1-^13^C]- and [2-^13^C]-ethanol, respectively. (D and E) Mass spectra of acetyl-CoA extracted from *A. flavus* mycelia of the control culture and culture supplemented with [1-^13^C]-ethanol, respectively. The observed mass and predicted molecular formula are shown above each peak. (F) Predicted pathway of ^13^C incorporation from labeled ethanol to aflatoxin B_1_. • and ∗ indicate ^13^C derived from ^13^C of [1-^13^C]- and [2-^13^C]-ethanol, respectively. See also Figure S2 and Tables S1 and S2.

To investigate the availability of ethanol in aflatoxin biosynthesis, *A. flavus* IFM 47798 was cultured with [1-^13^C]- or [2-^13^C]-ethanol for 48 h, and the aflatoxin B_1_ and B_2_ produced were analyzed by liquid chromatography/mass spectrometry (LC/MS). In samples cultured with [1-^13^C]-ethanol, nine ion peaks from *m/z* 313.07 to *m/z* 322.10 at intervals of 1.003, corresponding to the exact mass difference between ^13^C and ^12^C, were clearly observed for aflatoxin B_1_ (C_17_H_12_O_6_); they were designated [M+H]^+^ to [M+H+9]^+^ (Figures 2A and 2B). The relative abundances of [M+H+8]^+^, [M+H+9]^+^, and the slightly detected [M+H+10]^+^ (*m/z* 323.10) were 24.9, 8.4, and 0.4, respectively (Table S1). Considering that naturally occurring ^13^C is present at an abundance of approximately 1.1%, the abundance of [M+H+10]^+^ was explainable by the natural ^13^C contributions from [M+H]^+^ to [M+H+9]^+^ (see Table S1 for calculation). [M+H+9]^+^ was much more abundant than estimated by the natural ^13^C contribution. These results indicate that up to nine carbon atoms of aflatoxin B_1_ can be ^13^C labeled by [1-^13^C]-ethanol. Similarly, in samples cultured with [1-^13^C]-ethanol, nine ion peaks from *m/z* 315.09 to *m/z* 324.12 at intervals of 1.003 were observed for aflatoxin B_2_ (C_17_H_14_O_6_; Figures S2A and S2B). The application of the same calculation as for aflatoxin B_1_ indicated that up to nine carbon atoms of aflatoxin B_2_ can be ^13^C labeled by [1-^13^C]-ethanol.

In samples cultured with [2-^13^C]-ethanol, seven ion peaks appeared for aflatoxin B_1_ and were designated [M+H]^+^ to [M+H+7]^+^ (Figure 2C). The relative abundances of [M+H+6]^+^, [M+H+7]^+^, and slightly detected [M+H+8]^+^ were 14.7, 3.9, and 0.4, respectively (Table S2). The abundance of [M+H+8]^+^ was reasonable, considering the natural ^13^C contributions of [M+H]^+^ to [M+H+7]^+^, but that of [M+H+7]^+^ was much greater than estimated (Table S2). For aflatoxin B_2_, seven ion peaks designated [M+H]^+^ to [M+H+7]^+^ were identified, and the [M+H+8]^+^ peak was not observed (Figure S2C). These results indicate that up to seven carbon atoms of aflatoxin B_1_ and B_2_ are ^13^C labeled by [2-^13^C]-ethanol.

Acetyl-CoA was extracted from the fungal mycelia cultured with [1-^13^C]-ethanol and analyzed by LC/MS. [M-H+1]^-^ was more abundant than [M-H]^-^, indicating that the ^13^C of [1-^13^C]-ethanol was incorporated into the acetyl moiety of acetyl-CoA, and that the ^13^C-labeled acetyl-CoA was used for aflatoxin B_1_ and B_2_ biosynthesis (Figures 2D–2F and S2D).

### Ethanol incorporation into acetyl-CoA and aflatoxin occurred at high rates

**Figure 3:**
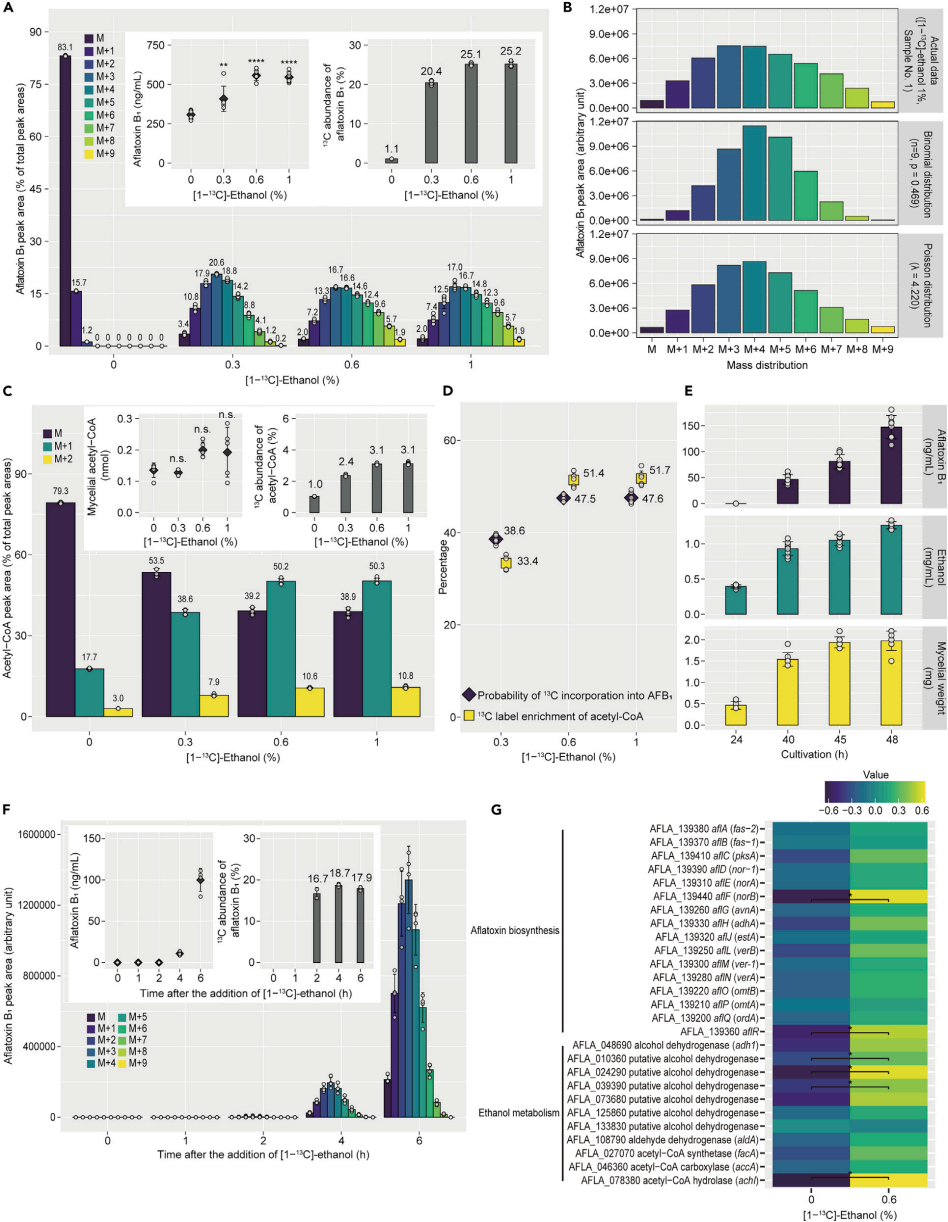
Added [1-^13^C]-ethanol was utilized for acetyl-CoA and aflatoxin B_1_ biosynthesis at high rates (A) Percentages of peak areas of aflatoxin B_1_ produced by *A. flavus* cultured with [1-^13^C]-ethanol; aflatoxin B_1_ concentrations and ^13^C abundance are shown in the inset panels. Mean ± SD, n = 6. ∗∗p< 0.01, ∗∗∗∗p< 0.0001 versus control, ANOVA followed by Dunnett’s test. (B) Mass distributions generated by binomial and Poisson distribution models with estimated parameters; upper, actual data from 1% [1-^13^C]-ethanol–treated sample #1; middle, peak areas calculated by binomial distribution with estimated *p* and n = 9; lower, peak areas from [M+H]^+^ to [M+H+9]^+^ calculated by Poisson distribution with estimated λ. (C) Percentages of peak areas of acetyl-CoA extracted from *A. flavus* cultured with [1-^13^C]-ethanol; acetyl-CoA quantities and ^13^C abundance are shown in the inset panels. Mean ± SD, n = 6. n.s., not significant, ANOVA followed by Dunnett’s test. (D) Probability of ^13^C incorporation into aflatoxin B_1_ and ^13^C label enrichment of acetyl-CoA, estimated by binomial regression and expressed as mole percent excess (MPE),^21^ respectively. Mean ± SD, n = 6. (E) Time-courses of *A. flavus* aflatoxin B_1_ production, ethanol production, and mycelial growth. Mean ± SD, n = 8. (F) Peak areas of aflatoxin B_1_ collected after the addition of 0.6% [1-^13^C]-ethanol at 24 h cultivation; aflatoxin B_1_ concentrations and ^13^C abundance are shown in the inset panels. Mean ± SD, n = 4. (G) Heatmap of relative gene expression patterns, determined by RT-qPCR 6 h after the addition of ethanol at 24 h cultivation. mRNA levels were standardized, and averages of n = 8 are shown. ∗p< 0.05, unpaired *t* test followed by the two-stage step-up procedure of Benjamini, Krieger, and Yeku to control the false discovery rate at 0.1. See also Figure S3.

To quantitatively evaluate the incorporation of ^13^C of labeled ethanol into aflatoxin B_1_, *A. flavus* was cultured with 0%, 0.3%, 0.6%, and 1% [1-^13^C]-ethanol for 48 h, and the aflatoxin B_1_ produced was analyzed by LC/MS (see Table 1 for mass range used for peak area quantification). As exhibited with unlabeled ethanol (Figure 1A), 0.3–1.0% [1-^13^C]-ethanol increased aflatoxin B_1_ production (Figure 3A, upper left panel). The ^13^C abundance in aflatoxin B_1_, which contains 17 carbon atoms, was also calculated using the peak area values (Figure 3A, upper right panel). For example, when relative peak areas from [M+H]^+^ to [M+H+9]^+^ were 2.0%, 7.4%, 12.6%, 17.0%, 16.7%, 14.8%, 12.3%, 9.6%, 5.7%, and 1.9%, respectively (as for 1% [1-^13^C]-ethanol in Figure 3A), the ^13^C abundance was 25.2% [(1 × 0.074 + 2 × 0.126 + 3 × 0.17 + 4 × 0.167 + 5 × 0.148 + 6 × 0.123 + 7 × 0.096 + 8 × 0.057 + 9 × 0.019) ÷ 17 × 100 = 25.2].^20^ Other isotopes, such as ^2^H and ^18^O, had limited abundance. [1-^13^C]-Ethanol at 0.3–1.0% also increased aflatoxin B_2_ production; the amount of aflatoxin B_2_ produced was approximately one-twelfth that of aflatoxin B_1_ (Figure S3A, upper left panel). With 0.3–1.0% [1-^13^C]-ethanol, the ^13^C abundance in aflatoxin B_2_ was 22.0–24.5%, comparable to that in aflatoxin B_1_ (Figure S3A, upper right panel).

When the contributions from natural isotopes are ignored and only ^13^C from labeled ethanol is considered, the mass distribution of aflatoxin B_1_ can be computed by the binomial distribution and Poisson distribution, the limiting case of binomial distribution.^22^ The peak area values from [M+H]^+^ to [M+H+9]^+^ of aflatoxin B_1_ were fitted to the binomial and Poisson distribution models, and the parameters were estimated. The mass distributions predicted by the two models with estimated parameters were strikingly close to the actual data (Figure 3B), supporting the assumption of binomial behavior of ^13^C incorporation from [1-^13^C]-ethanol into aflatoxin B_1_. The probability estimated with the binominal distribution model, taken as the probability of ^13^C incorporation into aflatoxin B_1_, was 38.6–47.6% (Figure 3D). Thus, this proportion of the aflatoxin B_1_ produced was likely [1-^13^C]-ethanol derived (158, 265, and 260 mg/mL for 0.3%, 0.6%, and 1% [1-^13^C]-ethanol, respectively). The increments in aflatoxin B_1_ caused by [1-^13^C]-ethanol (101, 250, and 239 mg/mL for 0.3%, 0.6%, and 1% [1-^13^C]-ethanol, respectively) were comparable to the amounts of [1-^13^C]-ethanol–derived aflatoxin B_1_, suggesting that the entire increase in aflatoxin B_1_ was attributable to the added ethanol.

The mycelial acetyl-CoA of *A. flavus* cultured with 0–1% [1-^13^C]-ethanol was extracted and analyzed by LC/MS.[1-^13^C]-ethanol did not significantly affect the amount of acetyl-CoA (Figure 3C, upper left panel), but resulted in an increase in the ^13^C abundance to 2.4–3.1% (Figure 3C, upper right panel). Based on the ratio of the peak area values of [M-H+1]^–^ and [M-H]^–^, the ^13^C label enrichment in acetyl-CoA was calculated to be 33.4–51.7% MPE (Figure 3D).^21^ The probability of ^13^C incorporation into aflatoxin B_1_ (38.6–47.6%) was comparable to the ^13^C label enrichment of acetyl-CoA, indicating that the rates of ^13^C incorporation into intracellular acetyl-CoA and aflatoxin B_1_ biosynthesis were consistent (Figure 3D).

*A. flavus* was cultured with 0.3–1.0% [2-^13^C]-ethanol, and the aflatoxin B_1_ and mycelial acetyl-CoA produced were analyzed. The ^13^C abundance in aflatoxin B_1_ was increased to 15.4–20.5%, less than with [1-^13^C]-ethanol, consistent with the finding that up to seven carbon atoms can be ^13^C-labeled by [2-^13^C]-ethanol (Figure S3B). For acetyl-CoA, the increase in ^13^C abundance was equivalent to that observed with [1-^13^C]-ethanol (Figure S3C). The probability of ^13^C incorporation into aflatoxin B_1_ was 37.5–49.4%, equivalent to that with [1-^13^C]-ethanol and comparable to the ^13^C label enrichment of acetyl-CoA (Figure S3D). Thus, there was no difference between the availability of [1-^13^C]-ethanol and [2-^13^C]-ethanol for acetyl-CoA and aflatoxin production.

To investigate the incorporation of ^13^C in a shorter time, 0.6% [1-^13^C]-ethanol was added 24 h after inoculation, when aflatoxin production had not started, and the aflatoxin B_1_ in the culture supernatant was analyzed 0, 1, 2, 4, and 6 h later. From the onset of aflatoxin production, the ^13^C abundance in aflatoxin B_1_ reached 16.7% and remained equivalent at 4 and 6 h (Figures 3E and 3F), indicating the ethanol is used for aflatoxin biosynthesis at a constant rate immediately after its addition. Fungal mRNA was collected from *A. flavus* exposed to ethanol for 6 h, and gene expression was examined by RT-qPCR. The gene expression of the aflatoxin production regulator *aflR*, aflatoxin biosynthetic enzyme *aflF*, and putative alcohol dehydrogenases AFLA_010360, AFLA_024290, and AFLA_039390 was significantly increased compared with the controls (Figure 3G), suggesting that ethanol stimulates the regulatory mechanisms of ethanol metabolism and aflatoxin production within a short time.

### Alcohol dehydrogenase *adh1* was involved in ethanol incorporation and aflatoxin production

**Figure 4:**
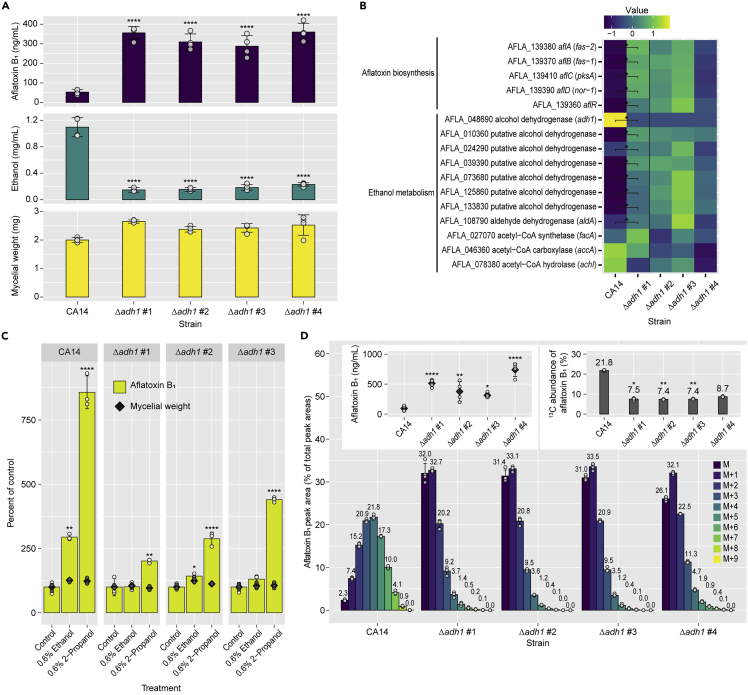
The alcohol dehydrogenase gene *adh1* plays primary roles in ethanol incorporation and aflatoxin production (A) Amounts of aflatoxin B_1_ and ethanol in the culture supernatant and mycelial dry weight of Δ*adh1* strains after 48 h cultivation. Mean ± SD, n = 6. ∗∗∗∗p< 0.0001 versus CA14, ANOVA followed by Dunnett’s test. (B) Heatmap of relative gene expression patterns, determined by RT-qPCR. Gene expression levels were standardized, and averages of n = 4 are shown. ∗p< 0.05, unpaired *t* test followed by the two-stage step-up procedure of Benjamini, Krieger, and Yeku to control the false discovery rate at 0.1. (C) Effects of ethanol and 2-propanol on the aflatoxin B_1_ production and mycelial growth of Δ*adh1* strains. The control values were set to 100% for each strain. Mean ± SD, n = 3. ∗p< 0.05, ∗∗p< 0.01, ∗∗∗∗p< 0.0001 versus each control, ANOVA followed by Dunnett’s test. (D) Percentages of peak areas of aflatoxin B_1_ produced by Δ*adh1* strains supplemented with [1-^13^C]-ethanol; aflatoxin B_1_ concentrations and ^13^C abundance are shown in the inset panels. Mean ± SD, n = 4. ∗p< 0.05, ∗∗p< 0.01, ∗∗∗∗p< 0.0001 versus CA14, ANOVA followed by Dunnett’s test (aflatoxin B_1_ concentration) or Kruskal–Wallis test followed by Dunn’s test (^13^C abundance). See also Figure S4.

Among the putative alcohol dehydrogenase genes in *A. flavus*, *adh1* encoding AFLA_048690 has the highest sequence identity (57% identity, 88% similarity) with yeast *adh1*, which plays a primary role in ethanol fermentation in yeast (Table S3).^23^ Thus, we prepared *adh1* deletion mutants using the *A. flavus* CA14 (Δ*ku70*Δ*pyrG*) strain, whose aflatoxin production was increased with the addition of 0.6% and 1% ethanol, as with the IFM 47798 strain (Figures S4A–S4D). The mycelial weights of Δ*adh1* strains cultured for 48 h were comparable to that of the parental strain, but the ethanol produced in the supernatant was decreased (Figure 4A). Aflatoxin B_1_ production was significantly increased in the Δ*adh1* strains. Gene expression in these strains was investigated by RT-qPCR, and was comparable to that in the parental strain (Figure 4B). Consistent with the increase in aflatoxin production, the expression of aflatoxin biosynthetic cluster genes was upregulated. All putative alcohol dehydrogenase genes apart from *adh1* and aldehyde dehydrogenase gene *aldA* (AFLA_108790) were also upregulated.

Δ*adh1* strains were incubated with 0.6% ethanol or 2-propanol, and aflatoxin B_1_ accumulation was quantified. Ethanol did not significantly affect aflatoxin production of Δ*adh1* strains #1 or #3. It increased the aflatoxin production of Δ*adh1* strain #2 by 1.4-fold, less than in the parental strain (2.9-fold). 2-Propanol increased aflatoxin production in the parental strain and all Δ*adh1* strains (Figure 4C). To confirm the involvement of *adh1* in the incorporation of ethanol into aflatoxin biosynthesis, CA14 and Δ*adh1* strains were cultured with 0.3% [1-^13^C]-ethanol and the aflatoxin B_1_ and B_2_ produced were analyzed. Aflatoxin B_1_ and B_2_ production remained higher in Δ*adh1* strains than in parental strains (Figure 4D and S4E, upper left panel), as was the case without ethanol (Figure 4A). The mass distribution patterns of aflatoxin B_1_ and B_2_ differed markedly between the parental and Δ*adh1* strains and the ^13^C abundance in aflatoxin B_1_ and B_2_ was significantly decreased in Δ*adh1* strains #1–3 compared with that in the parental strain (Figure 4D and Figure S4E, upper right panel), indicating that ^13^C incorporation into aflatoxin B_1_ and B_2_ from [1-^13^C]-ethanol was impaired by *adh1* gene deletion.

### *A. flavus* incorporated 2-propanol into acetyl-CoA and aflatoxin biosynthesis

To examine whether 2-propanol, like ethanol, is incorporated into aflatoxin biosynthesis, *A. flavus* was cultured with [2-^13^C]-2-propanol and the aflatoxin B_1_ and B_2_ and mycelial acetyl-CoA produced were analyzed by LC/MS.

**Figure 5:**
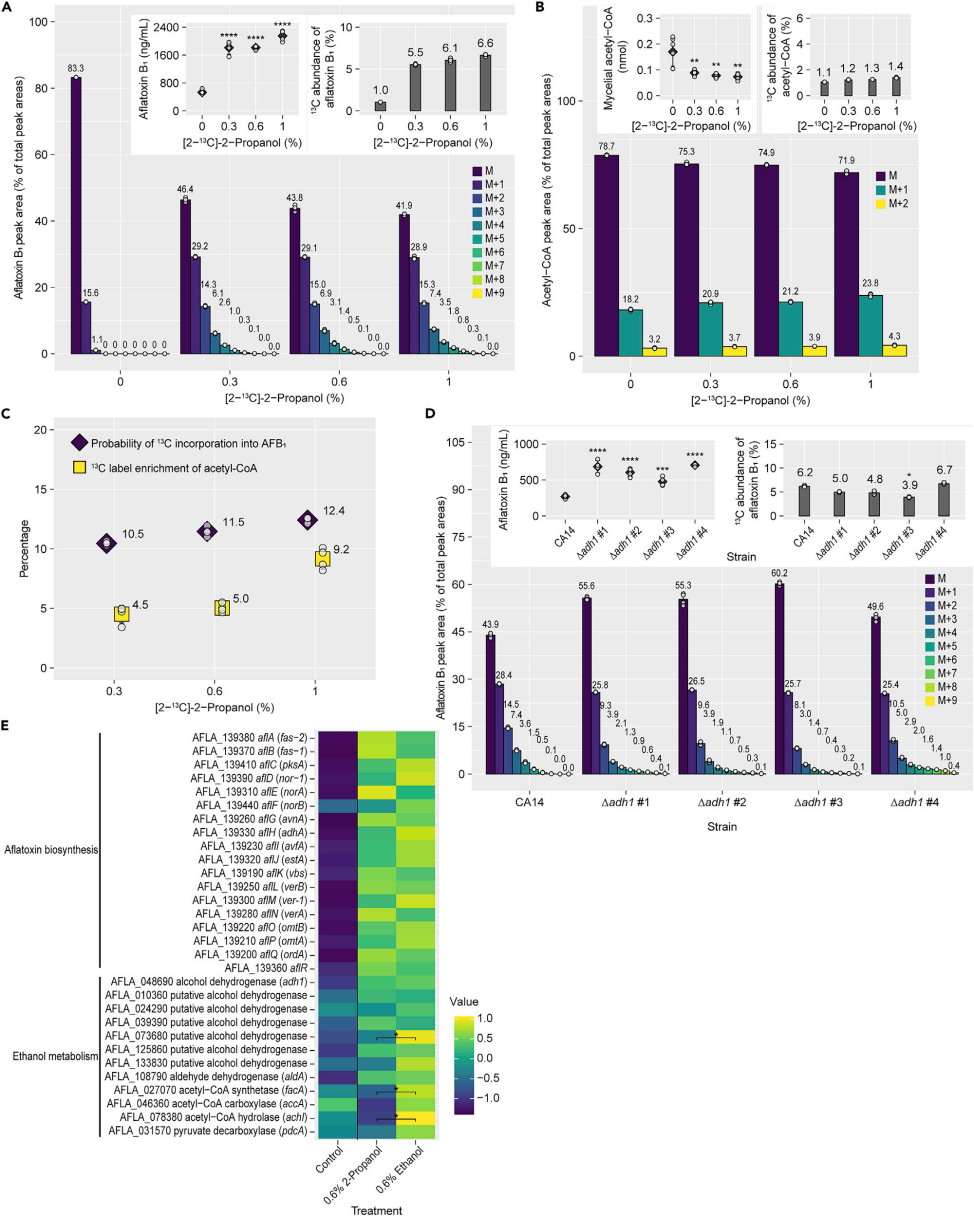
2-Propanol is inefficiently incorporated into acetyl-CoA and aflatoxin biosynthesis through a different pathway from that of ethanol (A) Percentages of peak areas of aflatoxin B_1_ from the culture supplemented with [2-^13^C]-2-propanol; aflatoxin B_1_ concentrations and ^13^C abundance are shown in the inset panels. Mean ± SD, n = 6. ∗∗∗∗p<0.0001 versus control, ANOVA followed by Dunnett’s test. (B) Percentages of peak areas of acetyl-CoA extracted from *A. flavus* cultured with [2-^13^C]-2-propanol; acetyl-CoA concentrations and ^13^C abundance are shown in the inset panels. Mean ± SD, n = 6. ∗∗p< 0.01 versus control, ANOVA followed by Dunnett’s test. (C) Probability of ^13^C incorporation into aflatoxin B_1_ and ^13^C label enrichment of fungal acetyl-CoA. Mean ± SD, n = 6. (D) Percentages of peak areas of aflatoxin B_1_ collected from the cultures of Δ*adh1* strains with 0.3% [2-^13^C]-2-propanol; aflatoxin B_1_ concentrations and ^13^C abundance are shown in the inset panels. Mean ± SD, n = 4. ∗p< 0.05, ∗∗∗p< 0.001, ∗∗∗∗p< 0.0001 versus CA14, ANOVA followed by Dunnett’s test (aflatoxin B_1_ concentrations) or Kruskal–Wallis test followed by Dunn’s test (^13^C abundance). (E) Heatmap of relative gene expression patterns, determined by RT-qPCR. Gene expression levels were standardized, and averages of n = 6 are shown. ∗p< 0.05, unpaired *t* test followed by the two-stage step-up procedure of Benjamini, Krieger, and Yeku to control the false discovery rate at 0.1. See also Figure S5.

The significant increase in aflatoxin B_1_ production attributable to [2-^13^C]-2-propanol was confirmed (Figure 5A, upper left panel). The mass distributions indicated that the ^13^C of [2-^13^C]-2-propanol was incorporated into aflatoxin B_1_, and the ^13^C abundance in the labeled aflatoxin B_1_ was 5.5–6.6% (Figure 5A, upper right panel). Aflatoxin B_2_ production was also increased by [2-^13^C]-2-propanol, and the ^13^C abundance in aflatoxin B_2_ was 5.5–7.0%, consistent with that in aflatoxin B_1_ (Figure S5A). Unexpectedly, [2-^13^C]-2-propanol decreased the amount of mycelial acetyl-CoA produced significantly (Figure 5B, upper left panel). The ^13^C abundance in acetyl-CoA increased to 1.2–1.4% with the addition of [2-^13^C]-2-propanol (Figure 5B, upper right panel), indicating that the ^13^C of [2-^13^C]-2-propanol was incorporated to acetyl-CoA. The probability of ^13^C incorporation into aflatoxin B_1_ and the ^13^C label enrichment of acetyl-CoA were comparable to each other, but much lower than those observed for [1-^13^C]-ethanol (Figure 5C).

To examine the involvement of *adh1* in the incorporation of 2-propanol into aflatoxin biosynthesis, Δ*adh1* strains were cultured with 0.3% [2-^13^C]-2-propanol and the aflatoxin B_1_ and B_2_ produced were analyzed (Figures 5D and S5B). The mass distributions of aflatoxin B_1_ and B_2_ in the Δ*adh1* strains resembled those in the parental strain. Consistent with this result, the ^13^C abundance in aflatoxin B_1_ and B_2_ did not differ between the parental and Δ*adh1* strains apart from Δ*adh1* #3, in which^13^C was slightly less abundant (Figures 5D and S5B, upper right panel). These results suggest that one or more alcohol dehydrogenases other than *adh1* is responsible for the incorporation of 2-propanol into the metabolic pathway of aflatoxin biosynthesis.

The effects of 2-propanol and ethanol on gene expression were compared using RT-qPCR. The aflatoxin biosynthetic cluster gene expression levels did not differ between the alcohols. The expression of a putative alcohol dehydrogenase gene, acetyl-CoA synthetase gene, and acetyl-CoA hydrolase gene was upregulated in ethanol (Figure 5E).

## Discussion

The use of alcohols for the development of an economical and effective method of preventing aflatoxin contamination of food and feed is promising.^10^ All low-molecular-weight alcohols tested in this study inhibited aflatoxin production at concentrations >2%, but ethanol, 2-propanol, and 2-methyl-2-propanol increased the aflatoxin B_1_ and B_2_ production of *A. flavus*IFM47798 at low concentrations. In accordance with the effects on aflatoxin production, 0.6% ethanol, 2-propanol, and 2-methyl-2-propanol increased the expression of most aflatoxin biosynthetic cluster genes. Thus, the application of these alcohols to prevent aflatoxin contamination may be counterproductive, as their non-uniform distribution on target crops could cause topical aflatoxin accumulation. We plan to investigate the applicability of methanol, 1-propanol, and 1-butanol as aflatoxin-controlling agents.

Consistent with previous reports on ^13^C- and ^14^C-labeled acetate incorporation,^18^^,^^19^ the C-1 and C-2 atoms of ethanol were incorporated into up to nine and seven carbon atoms, respectively, of aflatoxin B_1_ and B_2_. Considering that the branching point of aflatoxin B_1_ and B_2_ in the biosynthetic pathway is versicolorin B desaturation, and that the pathways before and after this desaturation are identical,^24^ the equivalence of the aflatoxin B_1_ and B_2_^13^C incorporation patterns is reasonable. Surprisingly, the addition of 0.6% and 1% [1-^13^C]-ethanol increased the ^13^C label enrichment of acetyl-CoA to levels exceeding 50%, meaning that >50% of the intracellular acetyl-CoA was ethanol derived, despite the synthesis of acetyl-CoA in various pathways in multiple organelles, such as β-oxidation in the mitochondria and peroxisome.^25^ Given the biosynthetic pathway from ethanol to aflatoxin B_1_, the ^13^C label enrichment of acetyl-CoA directed to aflatoxin biosynthesis should be equal to the probability of ^13^C incorporation into aflatoxin B_1_, as the amount of aflatoxin B_2_ was negligible compared with that of aflatoxin B_1_. Although the conversion of ethanol to acetyl-CoA may occur in the cytosol and the early steps of aflatoxin biosynthesis (acetyl-CoA conversion to norsolorinic acid) have been proposed to occur in the peroxisome,^25^^,^^26^^,^^27^ the ^13^C label enrichment of acetyl-CoA in whole cell extracts was equivalent to the probability of ^13^C incorporation into aflatoxin B_1_. This finding suggests that ethanol-derived acetyl-CoA is transported uniformly throughout the cell, including to the peroxisome, and is directed in an unbiased manner to aflatoxin biosynthesis. Furthermore, the ^13^C abundance in aflatoxin B_1_ reached nearly 17%, even with the addition of [1-^13^C]-ethanol just 2 h before the onset of aflatoxin accumulation, suggesting that ethanol-derived acetyl-CoA spreads in the cell immediately after the addition of ethanol, and that the biosynthesis of the aflatoxin B_1_ molecule from acetyl-CoA is accomplished within 2 h. To elucidate the intracellular source of acetyl-CoA and the kinetics of its utilization in aflatoxin biosynthesis, it would be beneficial to visualize and continuously observe the subcellular localization of ^13^C-labeled acetyl-CoA derived from ^13^C-labeled ethanol.

In Δ*adh1* strains, the effects of exogenous ethanol on the increases in the amount and ^13^C abundance in aflatoxin B_1_ and B_2_ were largely reduced, indicating that *adh1* is responsible for ethanol catabolism to acetyl-CoA and aflatoxin. Moreover, whereas ethanol production was ceased, aflatoxin biosynthetic cluster gene expression and aflatoxin B_1_ production were upregulated strongly in the Δ*adh1* strains. These results demonstrate the existence of a metabolic link between aflatoxin production and alcohol fermentation, for which*adh1* is primarily responsible. *adh1* may regulate this metabolic balance by determining the fate of acetaldehyde (i.e., whether it is converted to ethanol or acetate used for aflatoxin production). *adh1* gene deletion may cause the accumulation of acetate and nicotinamide adenine dinucleotide hydride (NADH), the reduced cofactor of alcohol dehydrogenases. As aflatoxin biosynthesis consumes many reducing agents in the form of nicotinamide adenine dinucleotide phosphate,^3^ the accumulation of NADH and acetate may lead to increased aflatoxin production. Considering that *adh1* gene is expressed under conditions conducive to aflatoxin production,^12^ and that ethanol production occurs before aflatoxin production, *A. flavus* may produce ethanol via *adh1* in advance, and metabolize it after the nutrient is depleted for use in aflatoxin biosynthesis.

We found that 2-propanol is also incorporated into aflatoxin biosynthesis via acetyl-CoA. As [2-^13^C]-2-propanol increased the ^13^C abundance in aflatoxin B_1_ and B_2_ in Δ*adh1* strains, *adh1* is not primarily responsible for 2-propanol catabolism; secondary alcohol dehydrogenases appear to be responsible for the oxidation of 2-propanol to acetone in *A. flavus*.^28^ Acetone is converted to methyl acetate by fungal Baeyer-Villiger–type monooxygenase^29^ and then decomposed into acetate, which is used for aflatoxin biosynthesis. Consistent with this view, Bennett et al.^15^ reported that 10 and 100 mM acetone increased the aflatoxin production of *A. parasiticus*. Although the probability of ^13^C incorporation into aflatoxin B_1_ and ^13^C label enrichment of acetyl-CoA were consistent for each [2-^13^C]-2-propanol concentration, these values were smaller than those for ethanol. Thus, although the ethanol-triggered increase in aflatoxin production may have resulted from the exploitation of ethanol as a carbon source, the hypothesized exploitation of 2-propanol as a carbon source does not explain the much greater increase in aflatoxin production caused by 2-propanol. Furthermore, unlike ethanol, 2-propanol decreased the amount of acetyl-CoA. Takasaki et al.^30^ reported that acetyl-CoA synthetase is required for the ethanol utilization pathway to acetyl-CoA via acetate in *Aspergillus nidulans*. Thus, the reduced expression of the acetyl-CoA synthetase gene in 2-propanol–treated *A. flavus* may be related to the decreased acetyl-CoA level. Further studies are needed to determine why 2-propanol promoted aflatoxin production more strongly than did ethanol, despite the reduced amount of acetyl-CoA and similarity of aflatoxin biosynthetic cluster gene expression between the alcohols.

Whether 2-methyl-2-propanol is also incorporated into aflatoxin biosynthesis in *A. flavus*, and whether ethanol and 2-propanol are incorporated into aflatoxin biosynthesis in *A. parasiticus*, remain elusive. Additional research is needed to investigate commonalities and differences in mechanisms of action among alcohols and aflatoxigenic fungi.

### Limitations of the study

In this study, we computed the probability of ^13^C incorporation into aflatoxin B_1_ by fitting the mass distribution of aflatoxin B_1_ to a binomial distribution model, although the Poisson distribution better approximated the real mass distribution data. This approach may have led to the oversimplification of the multitude of biosynthetic reactions that occur simultaneously in the cell. The neglect of the contribution of naturally occurring ^13^C in model fitting may have led to the overestimation of the probability of incorporation. The data we provide on the ^13^C label enrichment of acetyl-CoA e are approximate; to increase accuracy, the skew correction factor for natural ^13^C should be considered in the calculation of the sample tracer/tracee ratio. To examine the role of *adh1* in ethanol and 2-propanol utilization, we prepared Δ*adh1* strains. However, the increased expression of other putative alcohol dehydrogenase genes in these strains suggests that these putative alcohol dehydrogenases complement the function of *adh1*. Thus, the precise determination of *adh1*’s function may require the combined application of other methods.

## STAR★Methods

### Key resources table

**Table:** 

REAGENT or RESOURCE	SOURCE	IDENTIFIER
**Chemicals, peptides, and recombinant proteins**		

[1-^13^C]-Ethanol	Cambridge Isotope Laboratories, Inc.	Cat#CLM-344
[2-^13^C]-Ethanol	Cambridge Isotope Laboratories, Inc.	Cat#CLM-130
[2-^13^C]-2-Propanol	Cambridge Isotope Laboratories, Inc.	Cat#CLM-4714
Aflatoxins Mixture Standard Solution(B1, B2, G1, G2 each 25 μg/ml Acetonitrile Solution)	FUJIFILM Wako Pure Chemical	Cat#018-24341
Alcohol Dehydrogenase	Sigma-Aldrich	Cat#A3263
Aldehyde Dehydrogenase	Sigma-Aldrich	Cat#A6338

**Deposited data**		

All data and original code	Mendeley Data	https://doi.org/10.17632/gpnjw5xxm7.1

**Experimental models: Organisms/strains**		

*Aspergillus flavus*: IFM47798 strain	Medical Mycology Research Center, Chiba University	N/A
*Aspergillus flavus*: MAFF111229 strain	The Research Center of Genetic Resources, National Agriculture and Food Research Organization	N/A
*Aspergillus parasiticus*: NRRL2999 strain	Biological Resource Center, National Institute of Technology and Evaluation	N/A
*Aspergillus flavus*: CA14 strain: Δ*ku70*Δ*pyrG*	Fungal Genetics Stock Center	N/A
*Aspergillus flavus*: CA14 Δ*adh1* strain: Δ*ku70*Δ*pyrG*Δ*adh1*::*pyrG*	This Paper	N/A

**Oligonucleotides**		

RT-PCR primers	See Table S3	N/A
Primers used for generation and verification of mutant strains	See Table S4	N/A

**Software and algorithms**		

GraphPad Prism version 9.3.1	GraphPad software	https://www.graphpad.com/
RStudio Version 1.4.1106	RStudio, PBC	https://www.rstudio.com/products/rstudio/
Xcalibur Qual Browser	Thermo Fisher Scientific	https://www.thermofisher.com/order/catalog/product/OPTON-30965

### Resource availability

#### Lead contact

Further information and requests for data and resources should be directed to and will be fulfilled by the Lead Contact, Tomohiro Furukawa (furukawat795@affrc.go.jp).

#### Materials availability

Fungal strains generated in this study will be provided from the lead contact and may require a completed Materials Transfer Agreements.

### Experimental model and subject details

#### Fungal strains and culture conditions

The *A. flavus* strain IFM 47798 (Medical Mycology Research Center, Chiba University, Chiba, Japan) was used as the experimental model throughout this study. The *A. parasiticus* strain NRRL 2999 (Biological Resource Center, National Institute of Technology and Evaluation, Tokyo, Japan) was used to investigate the effect of alcohol on aflatoxin production. The *A. flavus* strain CA14 (Δ*ku70* Δ*pyrG*; Fungal Genetics Stock Center, Manhattan, KS, USA) was used as the parental strain in the construction of *adh1* gene deletion mutants (Δ*ku70* Δ*pyrG* Δ*adh1*::*pyrG*). Spores of these strains were collected from a 1-week-old culture plate, suspended in 30% glycerol solution, and stored at −80°C. The spore suspension was inoculated into potato dextrose broth liquid medium (Difco, Sparks, MD, USA) in a 12-well microplate (2 mL/well) at a density of 5 × 10^4^ spores/mL, and the microplate was placed at 28°C in the dark for the desired cultivation period. The incubation of the CA14 strain was conducted with uracil and uridine supplementation (1 mg/mL). Where indicated, alcohols were supplemented at the desired concentrations and cultivation timepoints. After cultivation, the culture broth was centrifuged to separate the supernatant and mycelia for subsequent analysis.

### Method details

#### Aflatoxin and acetyl-CoA extraction

To extract the aflatoxins produced, 0.5 mL culture supernatant was mixed with an equal amount of chloroform, and the chloroform solution was collected and evaporated by air drying. The remaining residue was dissolved in 1 mL 90% aqueous acetonitrile and subjected to LC/MS analysis. For acetyl-CoA extraction, the harvested fungal mycelia were washed and lyophilized. The dried mycelia were transferred to a Lysing matrix C tube (MP Biomedicals, Irvine, CA, USA) and ground (FastPrep-24; MP Biomedicals). Trifluoroacetic acid (5%; 200 μL) was added to the cell debris, with vigorous mixing at 4°C. After centrifugation at 10,000 × *g*, 20 μL 25% ammonia aqueous solution was added and mixed to neutralize, and then centrifuged at 15,000 × *g*. The supernatant was filtered and subjected to LC/MS analysis.

#### LC/MS analysis

The detection and quantification of the aflatoxins and acetyl-CoA were performed with an LC-Orbitrap MS Exactive system (Thermo Fisher Scientific, Waltham, MA, USA). The system was operated in accurate-mass/high-resolution full-scan mode at ultra-high resolution (100,000 full width at half maximum at *m/z* 200), in positive ion mode for aflatoxins and negative mode for acetyl-CoA. Parameters with the heated electrospray interface (ESI) were: sheath gas/aux gas/sweep gas, 30/5/0 arbitrary units; capillary temperature/heater temperature, 250°C/250°C; and spray voltage, 4.0 kV (positive) or −4.0 kV (negative). The capillary, tube lens, and skimmer voltages were set with the auto-tuning function for each run sequence. Mass calibration of the instrument was performed before each run sequence using calibration solutions [positive, Pierce LTQ Velos ESI Positive Ion Calibration Solution (Thermo Fisher Scientific); negative, Pierce Negative Ion Calibration Solution (Thermo Fisher Scientific)]. Chromatographic separation was performed on a 250 × 4.6 mm i.d. Capcell pak C18 UG120 column (Osaka Soda, Osaka, Japan) at 40°C. For the aflatoxins, the column was eluted with carrier solvents consisting of 0.1% formic acid (A) and acetonitrile (B), at the flow rate of 0.45 mL/min with a linear gradient of 5–95% B to 22 min. The retention times for aflatoxins B_1_, B_2_, G_1_, and G_2_ were 18.1, 17.6, 17.5, and 16.9 min, respectively. For acetyl-CoA, the solvents were composed of 5 mM hexylamine (pH 6.3; A) and 90% methanol/10% 10 mM ammonium acetate buffer (pH 8.5; B). Elution was conducted at the flow rate of 0.45 mL/min with a linear gradient of 5–95% B to 30 min. The retention time was 23.9 min. Table 1 shows the analyte ion masses and mass range used for peak area quantification for the target ions. The total of peak area values from [M+H]^+^ to [M+H+9]^+^ was used to calculate aflatoxin concentrations. Calibration curves were determined using an Aflatoxins Mixture Standard (containing 25 μg/mL B_1_, B_2_, G_1_, and G_2_; FUJIFILM Wako Pure Chemical, Osaka, Japan).

**Table 1:** Masses and mass ranges used for target ion quantification.

Compound	Formula	Ion name	Calculated mass (*m/z*)^a^	Mass range for quantification (*m/z*)^b^
Aflatoxin B_1_	C_17_H_12_O_6_	[M+H]^+^	313.0707	313.0691–313.0723
	C_16_^13^CH_12_O_6_	[M+H+1]^+^	314.0740	314.0724–314.0756
	C_15_^13^C_2_H_12_O_6_	[M+H+2]^+^	315.0774	315.0758–315.0790
	C_14_^13^C_3_H_12_O_6_	[M+H+3]^+^	316.0807	316.0791–316.0823
	C_13_^13^C_4_H_12_O_6_	[M+H+4]^+^	317.0841	317.0825–317.0857
	C_12_^13^C_5_H_12_O_6_	[M+H+5]^+^	318.0874	318.0858–318.0890
	C_11_^13^C_6_H_12_O_6_	[M+H+6]^+^	319.0908	319.0892–319.0924
	C_10_^13^C_7_H_12_O_6_	[M+H+7]^+^	320.0941	320.0925–320.0957
	C_9_^13^C_8_H_12_O_6_	[M+H+8]^+^	321.0975	321.0959–321.0991
	C_8_^13^C_9_H_12_O_6_	[M+H+9]^+^	322.1009	322.0993–322.1025
Aflatoxin B_2_	C_17_H_14_O_6_	[M+H]^+^	315.0863	315.0847–315.0879
	C_16_^13^CH_14_O_6_	[M+H+1]^+^	316.0895	316.0881–316.0913
	C_15_^13^C_2_H_14_O_6_	[M+H+2]^+^	317.0930	317.0914–317.0946
	C_14_^13^C_3_H_14_O_6_	[M+H+3]^+^	318.0964	318.0948–318.0980
	C_13_^13^C_4_H_14_O_6_	[M+H+4]^+^	319.0997	319.0981–319.1013
	C_12_^13^C_5_H_14_O_6_	[M+H+5]^+^	320.1031	320.1015–320.1047
	C_11_^13^C_6_H_14_O_6_	[M+H+6]^+^	321.1064	321.1048–321.1080
	C_10_^13^C_7_H_14_O_6_	[M+H+7]^+^	322.1098	322.1082–322.1114
	C_9_^13^C_8_H_14_O_6_	[M+H+8]^+^	323.1132	323.1116–323.1148
	C_8_^13^C_9_H_14_O_6_	[M+H+9]^+^	324.1165	324.1149–324.1181
Aflatoxin G_1_	C_17_H_12_O_7_	[M+H]^+^	329.0656	329.0640–329.0672
	C_16_^13^CH_12_O_7_	[M+H+1]^+^	330.0689	330.0672–330.0706
Aflatoxin G_2_	C_17_H_14_O_7_	[M+H]^+^	331.0812	331.0795–331.0829
	C_16_^13^CH_14_O_7_	[M+H+1]^+^	332.0846	332.0829–332.0863
Acetyl-CoA	C_23_H_37_O_17_N_7_P_3_S	[M-H]^-^	808.1174	808.1186–808.1234
	C_22_^13^CH_37_O_17_N_7_P_3_S	[M-H+1]^-^	809.1208	809.1216–809.1264
	C_21_^13^C_2_H_37_O_17_N_7_P_3_S	[M-H+2]^-^	810.1242	810.1254–810.1302

Aflatoxins were detected as hydrogen adducts in positive mode; acetyl-CoA was detected as hydrogen loss in negative mode.

a Calculated using Xcalibur Qual Browser software (Thermo Fisher Scientific).

b Peak areas of the target ions were quantified by setting the detection mass range in Xcalibur Qual Browser software.

#### Analysis of LC/MS data

See the Quantification and statistical analysis section below.

#### RT-qPCR

The sequences of the genes related to ethanol metabolism and production in *A. flavus* were determined from the JCVI-afl1-v2.0 genome assembly for *A. flavus* NRRL 3357 with database version 105.2 (http://fungi.ensembl.org/Aspergillus_flavus/Info/Index). A search for homologous protein sequences was conducted using the *Saccharomyces cerevisiae* proteins registered in the Uniprot database (https://www.uniprot.org/) as queries. The search was performed with Genetyx (Tokyo, Japan) software. The sequences of aflatoxin biosynthetic cluster and 18S rRNA genes were obtained from the database. Primers for RT-qPCR were designed using Primer Express software (Thermo Fisher Scientific); their sequences are provided in Table S3.

For the preparation of cDNA, lyophilized *A. flavus* mycelia were ground using the FastPrep-24 instrument as described above. Total RNA was extracted using Trizol reagent (Thermo Fisher Scientific) and purified with the PureLink RNA Mini Kit (Thermo Fisher Scientific). cDNA was synthesized with ReverTra Ace qPCR RT Master Mix (TOYOBO, Osaka, Japan). qPCR was conducted using PowerUp SYBR Green Master Mix (Thermo Fisher Scientific) in a final volume of 25 μL for each reaction in an QuantStudio 12K Flex Real-Time PCR system (Thermo Fisher Scientific). The mRNA levels for each gene were normalized to those of control 18S rRNA genes for each sample. Then, the mRNA levels were standardized so that the average value was 0 and the variance was 1 in all samples for each gene. Using these standardized values, a heatmap was created.

#### Generation of *adh1* gene deletion mutants

The Δ*adh1* strains were prepared using the split-marker approach as described previously.^31^^,^^32^ The orotidine-5′-monophosphate decarboxylase (*pyrG*) gene, cloned from the genome of *A. flavus* strain IFM 47798, was introduced into the *adh1* locus of the genome of *A. flavus* strain CA14 (Δ*ku70* Δ*pyrG*) for transformant selection based on uracil/uridine auxotrophy of the CA14 strain. Figure S3C is a schematic diagram of gene deletion. Sequences of the primers used for the preparation of replacement constructs and verification of gene deletion are listed in Table S4. To generate replacement constructs, the 5′and-3′ flanking regions of the *adh1* gene were amplified in a first-round PCR with the primer pairs Del_1F/Del_2R and Del_3F/Del_4R, respectively, and named amplicons 1 and 2. Next, the *pyrG* gene in the genome of *A. flavus* strain IFM 47798 was amplified with the primer pair *pyrG*P5F/*pyrG* P6R and named amplicon 3. The PCR products were purified using the QIAquick PCR purification kit (QIAGEN, Venlo, The Netherlands). In a second-round PCR, replacement constructs 1 and 2 were generated with the primer pair Del_1F/YR-R using amplicons 1 and 3 as templates, and with the primer pair PY-F/Del_4R using amplicons 2 and 3 as templates. The resulting replacement constructs 1 and 2 were introduced into the protoplasts of *A. flavus* strain CA14, and transformation mixture was spread onto selection plates without uracil or uridine. Candidate *adh1* deletion strains were screened based on uracil or uridine autotrophy of transformants. The desired gene deletion was verified by PCR with the primer pairs Check_1F/Check_1R and Check_2F/Check 2R (Figures S3D and S3E).

#### Ethanol quantification

The supernatant obtained by *A. flavus* culture centrifugation was diluted 10-fold in water. The diluted solution (10 μL) was transferred to a tube containing 250 μL enzyme solution [0.8 mg/mL β-nicotinamide adenine dinucleotide (Sigma-Aldrich), 0.06 mg/mL alcohol dehydrogenase (Sigma-Aldrich), 0.03 mg/mL aldehyde dehydrogenase (Sigma-Aldrich), 20 mM potassium phosphate (pH 8.0)]. The oxidation of ethanol to acetic acid was accompanied by the reduction of NAD^+^ to NADH, which has absorption at 340 nm. Following incubation at room temperature for 30 min, absorbance at 340 nm was measured. Ethanol concentrations were determined from the standard curve of an ethanol dilution series.

### Quantification and statistical analysis

#### Estimation of the probability of ^13^C incorporation into aflatoxin B_1_ from LC/MS data

For estimation, the presences of stable isotopes other than ^13^C and natural ^13^C was ignored due to the limited abundance of these isotopes relative to that of incorporated ^13^C from labeled alcohol. When the contribution of ^13^C from ^13^C-labeled alcohols alone is taken into account, the mass distribution of aflatoxin B_1_ should be computed using the binomial distribution formula $p\left(k,n\right)=\left(\begin{array}{l} n\\ k \end{array}\right)p^{k}{\left(1-p\right)}^{n-k}$, where parameter *n* is the maximum number of carbon atoms that may be ^13^C labeled, *k* is the number of ^13^C-labeled carbon atoms, and parameter *p* is the probability of success.^22^ The parameter *n* was set to 9 for the [1-^13^C]-ethanol– and [2-^13^C]-2-propanol–treated groups and to 7 for the [2-^13^C]-ethanol–treated group. If the probability *p* is small, *n* is large, and *np* is constant, the binomial distribution is approximated by Poisson distribution: $P\left(k\right)=\frac{\lambda^{k}}{k!}e^{-\lambda }$, where *k* is the number of ^13^C-labeled carbon atoms, parameter λ is the average number of ^13^C-labeled carbon atoms. The λ is approximately equal to *np*. Peak area values of aflatoxin B_1_ from [M+H]^+^ to [M+H+9]^+^ for [1-^13^C]-ethanol and [2-^13^C]-2-propanol and from [M+H]^+^ to [M+H+7]^+^ for [2-^13^C]-ethanol were subjected to binomial logistic regression using the GLM function in the R statistical platform (https://www.r-project.org). The binomial probability of success (*p*) was estimated using the maximum likelihood method and regarded as the probability of ^13^C incorporation into aflatoxin B_1_ in this study. Poisson regression was also performed using the GLM function in R to estimate the parameter λ.

#### Estimation of the ^13^C label enrichment of acetyl-CoA from LC/MS data

The ^13^C label enrichment of acetyl-CoA (in MPE) was calculated using the peak area values of [M-H]^–^ and [M-H+1]^–^ using an equation published previously.^21^^,^^33^ Briefly, the background tracer/tracee ratio (TTR; [M-H+1]^–^/[M-H]^–^) was calculated from control (no ^13^C-labeled alcohol treatment) peak area data. Similarly, the sample TTR was calculated as [M-H+1]^–^/[M-H]^–^ from ^13^C-labeled alcohol-treated sample peak area data. Then, TTR was calculated as TTR = sample TTR – average of background TTR. Finally, the MPE was calculated as MPE = TTR/(1 + TTR) × 100. MPE values reflect molecular enrichment, namely the percentage of molecules containing a labeled atom,^33^ and thus were regarded as reflecting the ^13^C label enrichment of acetyl-CoA.

#### Statistical analysis and graph creation

The data are presented as means ± standard deviations. For all quantitative data, the numbers of biological replicates (*n*s) used are provided in the relevant figure legends. GraphPad Prism ver. 9.3.1 (GraphPad Software, San Diego, CA, USA) was used to perform all statistical tests. Aflatoxin and ethanol quantities were compared between groups using the two-tailed Welch’s *t* test, and among more than two groups using one-way ANOVA with the post-hoc two-tailed Dunnett’s test. The ^13^C abundance in aflatoxin was compared using the nonparametric Kruskal–Wallis test with the post-hoc Dunn’s multiple comparisons test. Values of p< 0.05 were considered to be significant. Significant differences in gene expression between groups were determined using the multiple unpaired *t* test and the false discovery rate approach; the two-stage step-up procedure of Benjamini, Krieger, and Yeku was used with the false discovery rate set at 0.1. RStudio ver. 1.4.1106 (https://www.rstudio.com/products/rstudio/) and ggplot2 package (https://ggplot2.tidyverse.org/) were used for graph and heatmap creation. For preparation and arrangement of visual presentations, Adobe Illustrator ver.26.0.1 (https://www.adobe.com/jp/products/illustrator.html) was used.

## Data Availability

• All data have been deposited at Mendeley Data and are publicly available as of the date of publication. The DOI is listed in the key resources table. • All original R code has been deposited at Mendeley Data and is publicly available as of the date of publication. The DOI is listed in the key resources table. • Any additional information required to reanalyze the data reported in this paper is available from the lead contact upon request.

## References

[1] Bennett J.W., Klich M. (2003). Mycotoxins. Clin. Microbiol. Rev..

[2] Mitchell N.J., Bowers E., Hurburgh C., Wu F. (2016). Potential economic losses to the US corn industry from aflatoxin contamination. Food Addit. Contam. Part A Chem. Anal. Control Expo. Risk Assess..

[3] Yabe K., Nakajima H. (2004). Enzyme reactions and genes in aflatoxin biosynthesis. Appl. Microbiol. Biotechnol..

[4] Yu J., Chang P.-K., Ehrlich K.C., Cary J.W., Bhatnagar D., Cleveland T.E., Payne G.A., Linz J.E., Woloshuk C.P., Bennett J.W. (2004). Clustered pathway genes in aflatoxin biosynthesis. Appl. Environ. Microbiol..

[5] Chanda A., Roze L.V., Kang S., Artymovich K.A., Hicks G.R., Raikhel N.V., Calvo A.M., Linz J.E. (2009). A key role for vesicles in fungal secondary metabolism. Proc. Natl. Acad. Sci. USA.

[6] Sheridan K.J., Dolan S.K., Doyle S. (2014). Endogenous cross-talk of fungal metabolites. Front. Microbiol..

[7] Klich M.A. (2007). *Aspergillus flavus*: the major producer of aflatoxin. Mol. Plant Pathol..

[8] Lam F.H., Ghaderi A., Fink G.R., Stephanopoulos G. (2014). Engineering alcohol tolerance in yeast. Science.

[9] Ma M., Liu Z.L. (2010). Mechanisms of ethanol tolerance in *Saccharomyces cerevisiae*. Appl. Microbiol. Biotechnol..

[10] Ren Y., Jin J., Zheng M., Yang Q., Xing F. (2019). Ethanol inhibits aflatoxin B_1_ biosynthesis in *Aspergillus flavus* by up-regulating oxidative stress-related genes. Front. Microbiol..

[11] Sanchis V., Vinas I., Roberts I.N., Jeenes D.J., Watson A.J., Archer D.B. (1994). A pyruvate decarboxylase gene from *Aspergillus parasiticus*. FEMS Microbiol. Lett..

[12] Woloshuk C.P., Payne G.A. (1994). The alcohol dehydrogenase gene *adh1* is induced in *Aspergillus flavus* grown on medium conducive to aflatoxin biosynthesis. Appl. Environ. Microbiol..

[13] Flipphi M., Mathieu M., Cirpus I., Panozzo C., Felenbok B. (2001). Regulation of the aldehyde dehydrogenase gene (*aldA*) and its role in the control of the coinducer level necessary for induction of the ethanol utilization pathway in *Aspergillus nidulans*. J. Biol. Chem..

[14] Gupta S.R., Prasanna H.R., Viswanathan L., Venkitasubramanian T.A. (1975). The effect of inorganic salts and some biologically important compounds on the incorporation of 1—14C acetate into aflatoxins by resting mycelia of *Aspergillus parasiticus*. Z. Lebensm. Unters. Forsch..

[15] Bennett J.W., Lee L.S., Gaar G.G. (1976). Effect of acetone on production of aflatoxins and versicolorin pigments by resting cell cultures of *aspergillus parasiticus*. Mycopathologia.

[16] Biollaz M., Büchi G., Milne G. (1970). Biosynthesis of the aflatoxins. J. Am. Chem. Soc..

[17] Cox R. (2014). Oxidative rearrangements during fungal biosynthesis. Nat. Prod. Rep..

[18] Maggon K.K., Gupta S.K., Venkitasubramanian T.A. (1977). Biosynthesis of aflatoxins. Bacteriol. Rev..

[19] Pachler K.G.R., Steyn P.S., Vleggaar R., Wessels P.L., Scott D.B. (1976). Carbon-13 nuclear magnetic resonance assignments and biosynthesis of aflatoxin B_1_ and sterigmatocystin. J. Chem. Soc. Perkin 1.

[20] Lee W.N., Byerley L.O., Bergner E.A., Edmond J. (1991). Mass isotopomer analysis: theoretical and practical considerations. Biol. Mass Spectrom..

[21] Kim I.-Y., Suh S.-H., Lee I.-K., Wolfe R.R. (2016). Applications of stable, nonradioactive isotope tracers in in vivo human metabolic research. Exp. Mol. Med..

[22] Sadygov R.G. (2018). Poisson model to generate isotope distribution for biomolecules. J. Proteome Res..

[23] Roze L.V., Chanda A., Laivenieks M., Beaudry R.M., Artymovich K.A., Koptina A.V., Awad D.W., Valeeva D., Jones A.D., Linz J.E. (2010). Volatile profiling reveals intracellular metabolic changes in *Aspergillus parasiticus: veA* regulates branched chain amino acid and ethanol metabolism. BMC Biochem..

[24] Yabe K., Nakamura Y., Nakajima H., Ando Y., Hamasaki T. (1991). Enzymatic conversion of norsolorinic acid to averufin in aflatoxin biosynthesis. Appl. Environ. Microbiol..

[25] Roze L.V., Chanda A., Linz J.E. (2011). Compartmentalization and molecular traffic in secondary metabolism: a new understanding of established cellular processes. Fungal Genet. Biol..

[26] Boubekeur S., Camougrand N., Bunoust O., Rigoulet M., Guérin B. (2001). Participation of acetaldehyde dehydrogenases in ethanol and pyruvate metabolism of the yeast *Saccharomyces cerevisiae*: role of yeast ACDHs. Eur. J. Biochem..

[27] Maggio-Hall L.A., Wilson R.A., Keller N.P. (2005). Fundamental contribution of β-oxidation to polyketide mycotoxin production in planta. Mol. Plant Microbe Interact..

[28] Sealy-Lewis H.M., Fairhurst V. (1995). Substrate specificity of nine NAD+-dependent alcohol dehydrogenases in *Aspergillus nidulans*. Microbiology.

[29] Ferroni F.M., Tolmie C., Smit M.S., Opperman D.J. (2016). Structural and catalytic characterization of a fungal Baeyer-Villiger monooxygenase. PLoS One.

[30] Takasaki K., Shoun H., Yamaguchi M., Takeo K., Nakamura A., Hoshino T., Takaya N. (2004). Fungal ammonia fermentation, a novel metabolic mechanism that couples the dissimilatory and assimilatory pathways of both nitrate and ethanol. J. Biol. Chem..

[31] Furukawa T., Katayama H., Oikawa A., Negishi L., Ichikawa T., Suzuki M., Murase K., Takayama S., Sakuda S. (2020). Dioctatin activates ClpP to Degrade mitochondrial components and inhibits aflatoxin production. Cell Chem. Biol..

[32] Gravelat F.N., Askew D.S., Sheppard D.C., Brand A.C., MacCallum D.M. (2012). Host-Fungus Interactions Methods in Molecular Biology.

[33] Wolfe R.R., Chinkes D.L. (2004).

