# Supplementary material for: Low-dose ethanol increases aflatoxin production due to the adh1-dependent incorporation of ethanol into aflatoxin biosynthesis
Source: iScience. 2023 Jan 23;26(2):106051. doi: 10.1016/j.isci.2023.106051 (PMC9932502; doi:10.1016/j.isci.2023.106051)
Supplement: Document S1. Figures S1–S5 and Tables S1–S4 [file mmc1.pdf]

iScience, Volume *26*

Supplemental information

Low-dose ethanol increases aflatoxin production due to the *adh1*-dependent incorporation of ethanol into aflatoxin biosynthesis

Tomohiro Furukawa, Masayo Kushiro, Hiroyuki Nakagawa, Hirofumi Enomoto, and Shohei Sakuda

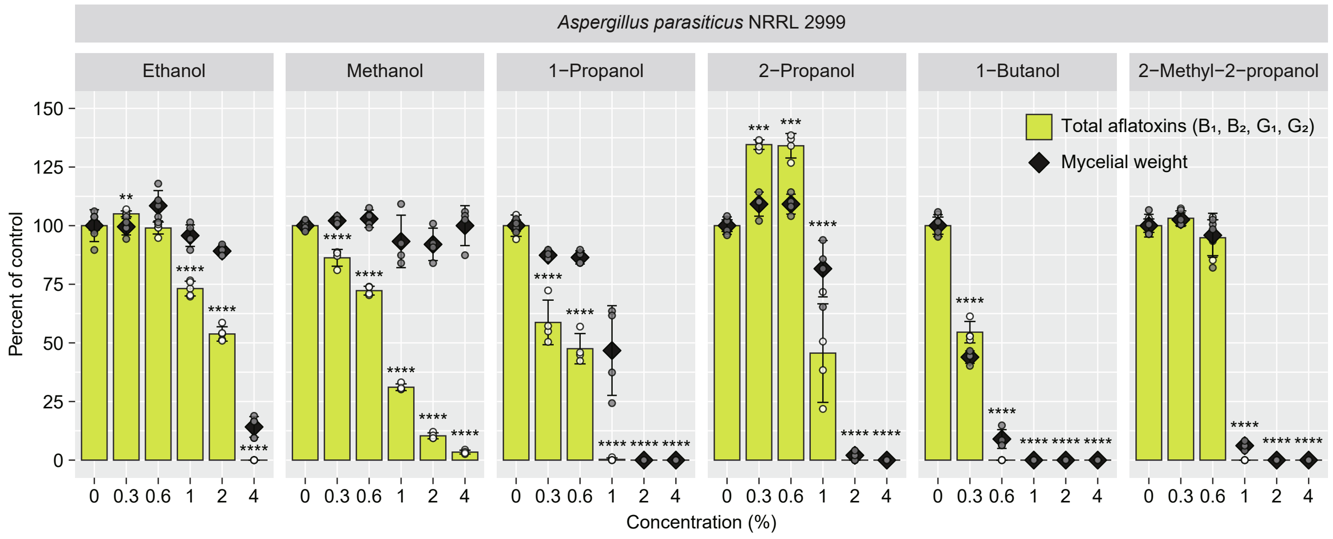

Figure S1 Effects of low-molecular-weight alcohols on the aflatoxins production and mycelial weight of *Aspergillus parasiticus* NRRL2999. Related to Figure 1. Mean ± SD, $n = 5$ (ethanol) or 4 (other alcohols), ANOVA followed by Dunnett's test. $^{**}p < 0.01$, $^{***}p < 0.001$, $^{****}p < 0.0001$, vs. control.

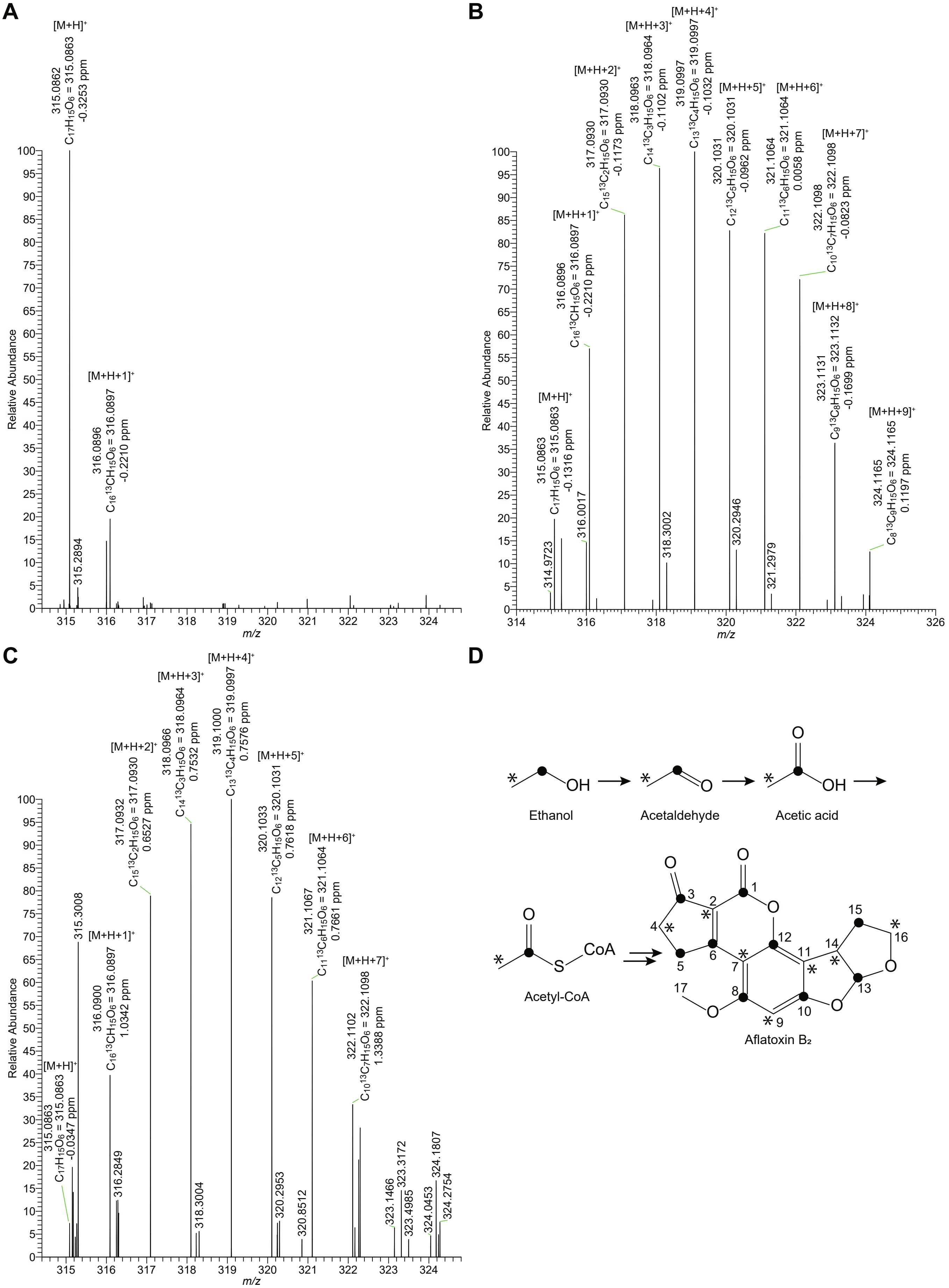

Figure S2 ^{13}C of [1-^{13}C]-ethanol and [2-^{13}C]-ethanol were also incorporation into aflatoxin B₂. Related to Figure 2.

(**A–C**) Mass spectra of aflatoxin B₂ extracted from control culture (**A**) and cultures supplemented with 1% of [1-^{13}C]- and [2-^{13}C]-ethanol, respectively (**B** and **C**). The observed mass, predicted molecular formula, and mass accuracy between theoretical and observed *m/z* are shown above each peak.

(**D**) Predicted pathway of ^{13}C incorporation from labeled ethanol to aflatoxin B₂.

● and * indicate ^{13}C derived from ^{13}C of [1-^{13}C]- and [2-^{13}C]-ethanol, respectively.

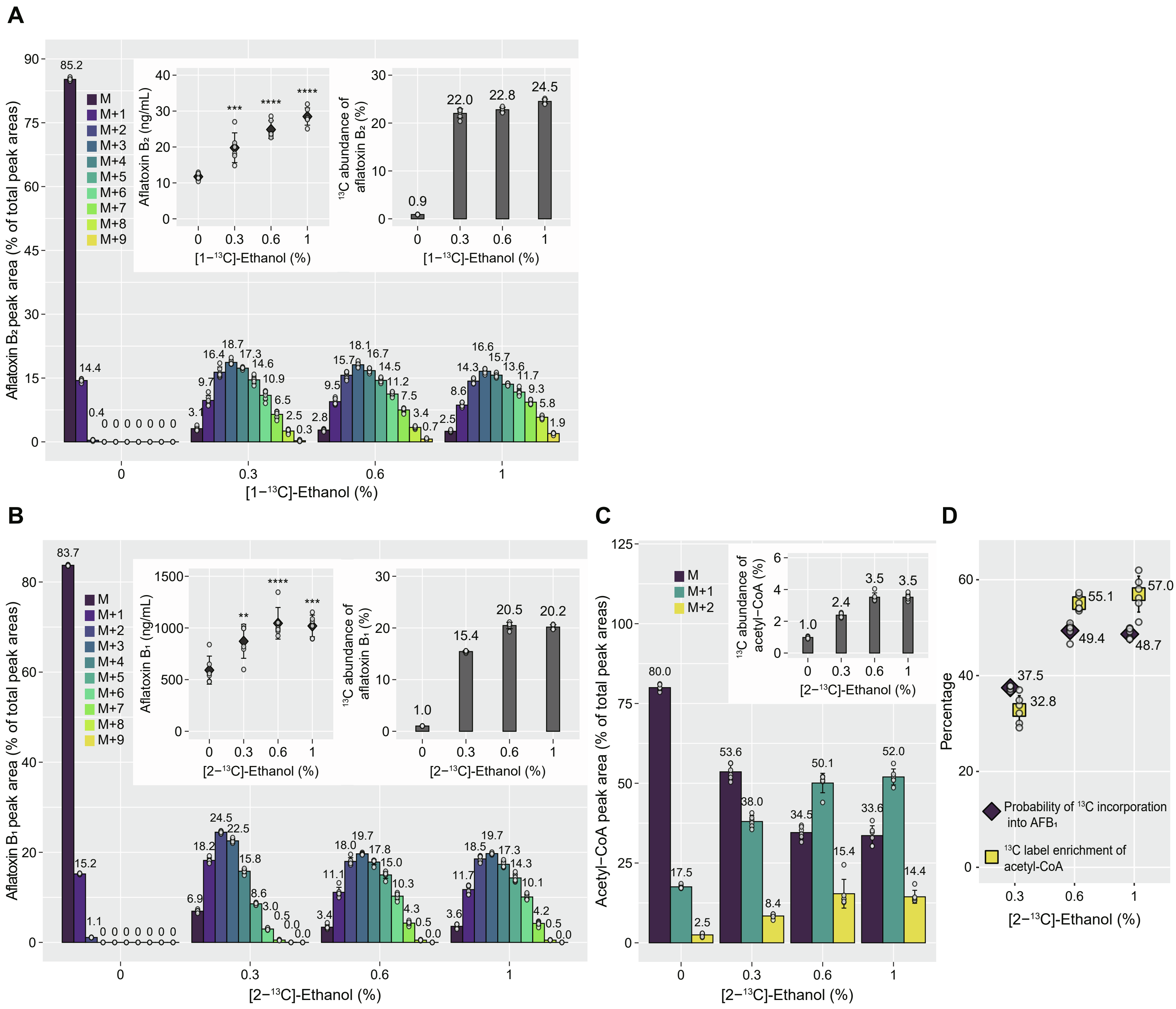

Figure S3 ^{13}C incorporation into aflatoxin B_1, aflatoxin B_2, and acetyl-CoA when labeled ethanol was added. Related to Figure 3.

(**A**) Percentages of peak areas of aflatoxin B_2 produced by *A. flavus* cultured with [1-^{13}C]-ethanol; aflatoxin B_2 concentrations and ^{13}C abundance are shown in the inset panels. Mean ± SD, $n = 6$. ***$p < 0.001$, ****$p < 0.0001$ vs. control, ANOVA followed by Dunnett's test.

(**B**) Percentages of peak areas of aflatoxin B_1 produced by *A. flavus* cultured with [2-^{13}C]-ethanol; aflatoxin B_1 concentrations and ^{13}C abundance are shown in the inset panels. Mean ± SD, $n = 6$. **$p < 0.01$, ***$p < 0.001$, ****$p < 0.0001$ vs. control, ANOVA followed by Dunnett's test.

(**C**) Percentages of peak areas of acetyl-CoA extracted from *A. flavus* cultured with [2-^{13}C]-ethanol; ^{13}C abundance shown in the inset panel. Mean ± SD, $n = 6$.

(**D**) Probability of ^{13}C incorporation into aflatoxin B_1 and ^{13}C label enrichment of acetyl-CoA, estimated by binomial regression and expressed as mole per excess percent excess (MPE), respectively. Mean ± SD, $n = 6$.

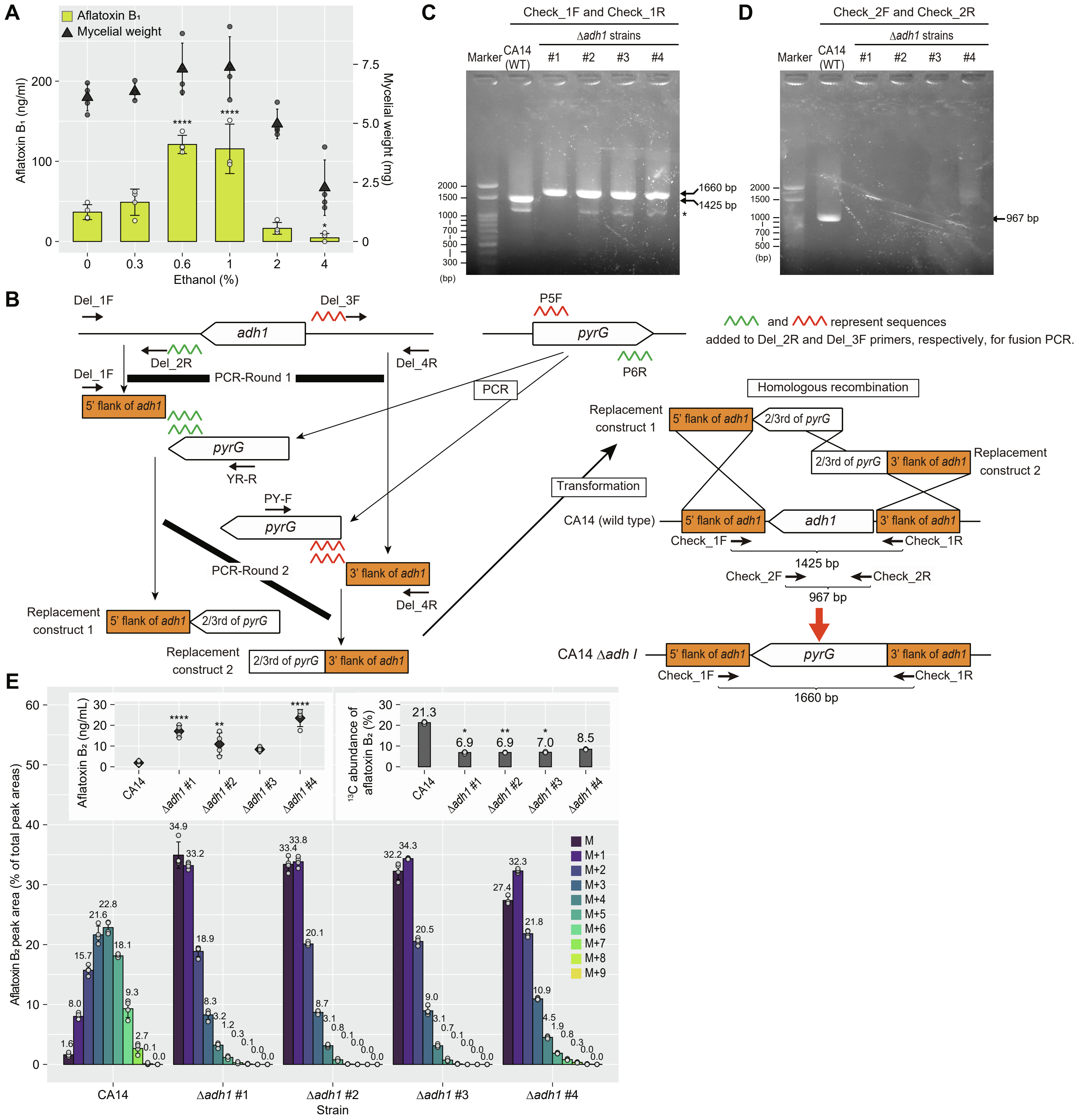

Figure S4 Preparation of deletion mutants of alcohol dehydrogenase gene *adh1* in CA14 strain. Related to Figure 4.

(**A**) Effects of ethanol on aflatoxin B_1 production and mycelial weight of *A. flavus* CA14.
Mean ± SD, $n = 4$. $*p < 0.05$, $****p < 0.0001$, vs. control, ANOVA followed by Dunnett's test.
(**B**) Schematic diagram of preparation of *adh1* gene deletion mutant. Orotidine-5'-monophosphate decarboxylase gene (*pyrG*) was used as the selective marker of deletion candidates, based on the uridine-dependent auxotroph of CA14 strain.
Locations of the primers are represented by black arrows. The sequences of primers are listed in Table S4.
(**C** and **D**) PCR verification of *adh1* gene deletion. Genomic DNAs extracted from CA14 and Δ*adh1* candidate strains were subjected to PCR using indicated primers. Asterisk represents non-specific PCR products.
(**E**) Percentages of peak areas of aflatoxin B_2 produced by Δ*adh1* strains supplemented with [1-^{13}C]-ethanol; aflatoxin B_2 concentrations and ^{13}C abundance are shown in the inset panels. Mean ± SD, $n = 4$. $*p < 0.05$, $**p < 0.01$, $****p < 0.0001$ vs. CA14, ANOVA followed by Dunnett's test (aflatoxin B_2 concentration) or Kruskal–Wallis test followed by Dunn's test (^{13}C abundance).

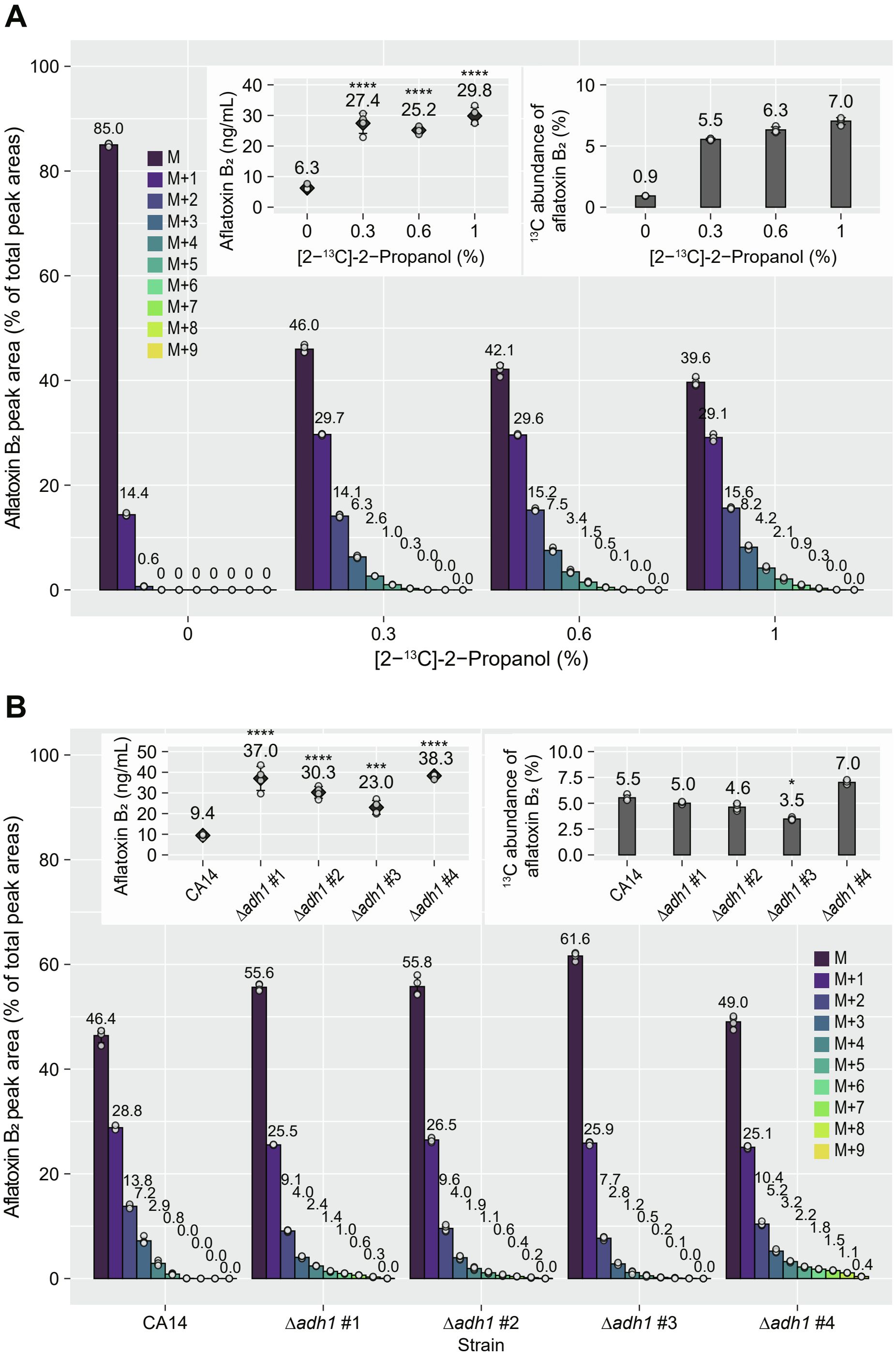

Figure S5 ^{13}C of [2-^{13}C]-2-Propanol was also incorporation into aflatoxin B_2. Related to Figure 5.

(**A**) Percentages of peak areas of aflatoxin B_2 produced by *A. flavus* cultured with [2-^{13}C]-2-propanol; aflatoxin B_2 concentrations and ^{13}C abundance are shown in the inset panels. Mean ± SD, $n = 6$ (control group) and 4 ([2-^{13}C]-2-propanol treated groups). ****$p < 0.0001$ vs. control, ANOVA followed by Dunnett's test.
(**B**) Percentages of peak areas of aflatoxin B_2 collected from the cultures of Δ*adh1* strains with 0.3% [2-^{13}C]-2-propanol; aflatoxin B_2 concentrations and ^{13}C abundance are shown in the inset panels. Mean ± SD, $n = 4$.
*$p < 0.05$, ***$p < 0.001$, ****$p < 0.0001$ vs. CA14, ANOVA followed by Dunnett's test (aflatoxin B_2 concentrations) or Kruskal–Wallis test followed by Dunn's test (^{13}C abundance).

Table S1. Estimation of ^{13}C incorporation into aflatoxin B_1 from [1-^{13}C]-ethanol, Related to Figure 2.

Observed *m/z*	313.070886	314.073687	315.077562	316.080631	317.084683	318.086868	319.089989	320.094021	321.09701	322.100844	323.103549
Formula	$C_{17}H_{13}O_6$	$C_{16}{}^{13}C_1H_{13}O_6$	$C_{15}{}^{13}C_2H_{13}O_6$	$C_{14}{}^{13}C_3H_{13}O_6$	$C_{13}{}^{13}C_4H_{13}O_6$	$C_{12}{}^{13}C_5H_{13}O_6$	$C_{11}{}^{13}C_6H_{13}O_6$	$C_{10}{}^{13}C_7H_{13}O_6$	$C_9{}^{13}C_8H_{13}O_6$	$C_8{}^{13}C_9H_{13}O_6$	$C_7{}^{13}C_{10}H_{13}O_6$
Label	$[M+H]^+$	$[M+H+1]^+$	$[M+H+2]^+$	$[M+H+3]^+$	$[M+H+4]^+$	$[M+H+5]^+$	$[M+H+6]^+$	$[M+H+7]^+$	$[M+H+8]^+$	$[M+H+9]^+$	$[M+H+10]^+$
Observed intensity	51953.23	151173.13	238743.33	262597.41	194023.50	176273.23	137345.19	102922.27	65428.24	21940.20	1164.91
Relative abundance	19.78	57.57	90.92	100.00	73.89	67.13	52.30	39.19	24.92	8.36	0.44
Contribution to the $[M+H+10]^+$ abundance[a]	0.00	0.00	0.00	0.00	0.00	0.00	0.00	0.01	0.11	0.74	0.86[b]
Contribution to the $[M+H+9]^+$ abundance[c]	0.00	0.00	0.00	0.00	0.00	0.00	0.01	0.22	2.49	2.72[d]	

[a]Assuming that $[M+H+10]^+$ is derived from the natural ^{13}C contribution, approximate abundance values for the contribution to $[M+H+10]^+$ from $[M+H]^+$ to $[M+H+9]^+$ were calculated. For example, the contribution from $[M+H+9]^+$ was calculated as follows: as natural ^{13}C is present at an approximate abundance of 1.1%, the abundance of $[M+H+10]^+$ ($C_7{}^{13}C_{10}H_{13}O_6$), in which one more ^{12}C atom of the remaining eight ^{12}C atoms of $[M+H+9]^+$ ($C_8{}^{13}C_9H_{13}O_6$) is replaced by natural ^{13}C, is estimated to be $\frac{\binom{8}{1}*0.011^1*0.989^7}{0.989^8} = 0.089$ when the abundance of $[M+H+9]^+$ is set to 1. Multiplied by the relative abundance of $[M+H+9]^+$, the contribution from $[M+H+9]^+$ to $[M+H+10]^+$ is estimated to be $8.36 \times 0.089 = 0.74$.

[b]Sum of the contributions from $[M+H]^+$ to $[M+H+9]^+$. The value is comparable to the observed relative abundance, indicating that $[M+H+10]^+$ can be attributed to the natural ^{13}C contribution from $[M+H]^+$ to $[M+H+9]^+$.

[c]Assuming that $[M+H+9]^+$ is derived from the natural ^{13}C contribution, approximate abundance values for the contribution to $[M+H+9]^+$ from $[M+H]^+$ to $[M+H+8]^+$ were calculated. For example, the contribution from $[M+H+7]^+$ was calculated as follows: the abundance of $[M+H+9]^+$, in which two more ^{12}C atoms of the 10 ^{12}C atoms of $[M+H+7]^+$ ($C_{10}{}^{13}C_7H_{13}O_6$) are replaced by natural ^{13}C, is estimated to be $\frac{\binom{10}{2}*0.011^2*0.989^8}{0.989^{10}} = 0.0056$ when $[M+H+7]^+$ is set to 1. Therefore, the contribution from $[M+H+7]^+$ to $[M+H+9]^+$ is calculated to be $39.19 \times 0.0056 = 0.22$.

[d]Sum of the contributions from $[M+H]^+$ to $[M+H+8]^+$. The value is much smaller than the observed abundance, indicating that $[M+H+9]^+$ cannot be explained by the natural ^{13}C contribution alone, i.e., $[M+H+9]^+$ was derived from ^{13}C incorporation from added ^{13}C.

Table S2. Estimation of ^{13}C incorporation into aflatoxin B_1 from [2-^{13}C]-ethanol, Related to Figure 2.

Observed *m/z*	313.071038	314.073814	315.077664	316.080716	317.083796	318.087854	319.090953	320.094004	321.097932
Formula	$C_{17}H_{13}O_6$	$C_{16}{}^{13}C_1H_{13}O_6$	$C_{15}{}^{13}C_2H_{13}O_6$	$C_{14}{}^{13}C_3H_{13}O_6$	$C_{13}{}^{13}C_4H_{13}O_6$	$C_{12}{}^{13}C_5H_{13}O_6$	$C_{11}{}^{13}C_6H_{13}O_6$	$C_{10}{}^{13}C_7H_{13}O_6$	$C_9{}^{13}C_8H_{13}O_6$
Label	$[M+H]^+$	$[M+H+1]^+$	$[M+H+2]^+$	$[M+H+3]^+$	$[M+H+4]^+$	$[M+H+5]^+$	$[M+H+6]^+$	$[M+H+7]^+$	$[M+H+8]^+$
Observed intensity	56696.72266	138612.5625	189037.8125	171276	122706.5234	67372.64844	27864.01172	7313.080078	783.017761
Relative abundance	29.99	73.33	100.00	90.60	64.91	35.64	14.74	3.87	0.41
Contribution to the $[M+H+8]^+$ abundance[a]	0.00	0.00	0.00	0.00	0.00	0.01	0.10	0.43	0.54[b]
Contribution to the $[M+H+7]^+$ abundance[c]	0.00	0.00	0.00	0.00	0.03	0.29	1.80	2.12[d]	

[a]Assuming that $[M+H+8]^+$ is derived from the natural ^{13}C contribution, approximate abundance values for the contribution to $[M+H+8]^+$ from $[M+H]^+$ to $[M+H+9]^+$ were calculated.

[b]Sum of the contributions from $[M+H]^+$ to $[M+H+7]^+$. The value is comparable to the observed relative abundance, indicating that $[M+H+8]^+$ can be attributed to the natural ^{13}C contribution from $[M+H]^+$ to $[M+H+7]^+$.

[c]Assuming that $[M+H+7]^+$ is derived from the natural ^{13}C contribution, approximate abundance values for the contribution to $[M+H+7]^+$ from $[M+H]^+$ to $[M+H+6]^+$ were calculated.

[d]Sum of the contributions from $[M+H]^+$ to $[M+H+6]^+$. The value is much smaller than the observed abundance, indicating that $[M+H+7]^+$ cannot be explained by the natural ^{13}C contribution alone, i.e., $[M+H+7]^+$ was derived from ^{13}C incorporation from added ^{13}C.

Table S3. Primers used for RT-qPCR, Related to STAR Methods.

Biological process	Gene (Yeast)	Protein (Yeast)	Identity (%)	Similarity (%)	EnsemblFungi ID (*A. flavus*)	EnsemblFungi Description (*A. flavus*)	Primer pair (5'→3')	
Ethanol biosynthetic process	ADH1	Alcohol dehydrogenase 1	57	88	AFLA_048690	alcohol dehydrogenase, putative	CAAGCCATGTGCGAGCAACT	TAGCGGCTTTGACATCTGCAA
			33	73	AFLA_010360	quinone oxidoreductase, putative	GGCGATTGGGTGGTGATTT	CCTTTATCCGAGCAGCAATCTG
			44	74	AFLA_024290	alcohol dehydrogenase, putative	ACGCAGGCGAGTCAAAGAGA	AGGATCGGACGTTTCGATGA
			42	72	AFLA_039390	alcohol dehydrogenase, putative	ACTCGACCTCCGCCATGATA	AGAAACCAGTGCATTCCAACTTG
			42	74	AFLA_073680	alcohol dehydrogenase, putative	ACGCCCAATCTGTGCAGTTC	GATGGGCTGAGGGTTGTTTTC
			46	78	AFLA_125860	alcohol dehydrogenase, putative	CCGCTCGTTTTGGCTTTG	CGCTTCCTCGACATCTCTCAA
			35	74	AFLA_133830	alcohol dehydrogenase, putative	GCCACCGCTTTCTTCGATT	CGAGCCCACCCGTTAGTTT
Ethanol catabolic process	ALD1	Aldehyde dehydrogenase 1, mitochondrial	38	73	AFLA_108790	aldehyde dehydrogenase AldA, putative	TGGCTGGGCCGATAAGATT	GTGGCGGGTGTAGGTAAGAGACT
Acetyl-CoA biosynthetic process from acetate	ACS1	Acetyl-coenzyme A synthetase 1	63	89	AFLA_027070	acetyl-coenzyme A synthetase FacA	CGCAAGTCGATTGGACCATT	ATCTTACCGCTGCGAGTCTTAGG
Malonyl-CoA biosynthetic process	ACC1	Acetyl-CoA carboxylase	64	89	AFLA_046360	acetyl-CoA carboxylase, putative	CACAGGTGCTCCGGCTATTAA	ATACATAATCTGCGTTCCACCAAGT
Acetate metabolic process	ACH1	Acetyl-CoA hydrolase	66	89	AFLA_078380	acetyl-coA hydrolase Ach1, putative	CACCCCGACTACAAGCCAAT	TCGTGGCCCATACCCTTCT
Glycolytic fermentation to ethanol	PDC1	Pyruvate decarboxylase isozyme 1	48	83	AFLA_031570	pyruvate decarboxylase PdcA, putative	AACCGGCACTGCCAATTTC	TACCCCAAAGAACCTGACTAATGG
Aflatoxin biosynthetic process					AFLA_139380	aflA / fas-2 / hexA / fatty acid synthase alpha subunit	TTGGCCGCCTCCTCTATTC	GGCTTGTCTCTCGTCATACTCATAAC
					AFLA_139370	aflB / fas-1 / fatty acid synthase beta subunit	GCATATTGTCGGACTGTCGAAGT	CAAACGAGAACCGTCCCTAAAG
					AFLA_139410	aflC / pksA / pksL1 / polyketide synthase	TGCATGGCGATGTGGTAGTT	GTAAGGCCGCGGAAGAAAG
					AFLA_139390	aflD / nor-1 / reductase	AGTCCAAGCAACAGGCCAAGT	CGCGCATAGTCGTGCATGT
					AFLA_139310	aflE/ norA/ aad/ adh-2/ NOR reductase/ dehydrogenase	TATCATCTAGCGCCGGTGTTC	CATTACCCCTTTCCAGCCATT
					AFLA_139440	aflF / norB / dehydrogenase	GCACATTTCACTGATGGATGAAG	CGGAAATCCAGGATCAAACTCA
					AFLA_139260	aflG/ avnA/ ord-1/ cytochrome P450 monooxygenase	GCAACCTTGTCCGCGATCT	ACAGCTCATTGGGTGCGATT
					AFLA_139330	aflH/ adhA/ short chain alcohol dehydrogenase	CAGGTAAACTTGATCGGCGTCTA	GATTGGCGGCCTTGTTTGT
					AFLA_139230	aflI/ avfA/ cytochrome P450 monooxygenase	TCAAATCCTCGTTCGGTCAAA	CGTGGACTTGTGGGTTTCTTG

					AFLA_139320	aflJ/ estA/ esterase	TGAGGAGAATGCGGAGTTGAC	ACCGGCGCCTTGTAACAAT
					AFLA_139190	aflK/ vbs/ VERB synthase	CAGATCGGAGCCAAGAAGGA	CCGATTCCAGACACCATTAGC
					AFLA_139250	aflL/ verB/ desaturase/ P450 monooxygenase	CAACAAGCGGATGAAAATGGA	GCTGATTATCGTCGGCACTCT
					AFLA_139300	aflM/ ver-1/ dehydrogenase/ ketoreductase	TGAGGCAACTGCGAAGTTAATG	CCAGCGTTCGATGACACGAT
					AFLA_139280	aflN/ verA/ monooxygenase	CTTTAGCGGGACCTGGATGA	TGGCCGGTTTGGTTAAATTC
					AFLA_139220	aflO/ omtB/ dmtA/ O-methyltransferase B	AGAGAGCGACACGCCGATAA	GAAGAATGCGACCAAGGAGTCT
					AFLA_139210	aflP/ omtA/ omt-1/ O-methyltransferase A	ACCAAGGAGTGGAATTCGCTTAT	GCCTTTGCGCCACCATATCT
					AFLA_139200	aflQ/ ordA/ ord-1/ oxidoreductase/ cytochrome P450 monooxigenase	AATCGGGATTCGACGGTTCTT	CGTTGGCTTCCTTTCGGATAT
					AFLA_139360	aflR / apa-2 / afl-2 / transcription activator	TCCGCCATCTTTTCTCATCA	CCGAATTCCGAATCGACTGTTA
					AFLA_139340	aflS/ pathway regulator	CGAGTCGCTCAGGCGCTCAA	GCTCAGACTGACCGCCGCTC
Ribosomal RNA					G4B84_011247	18S ribosomal RNA	CTCACCAGGTCCAGACAAAATAAGG	GCACCACCATCCAAAAGATCA

Table S4. Primers used for preparation of Δ*adh1* strains, Related to STAR Methods.

Application	Primer name	Sequence (5'→3')	Notes
Amplification of 5' flank region and preparation of replacement construct 1	Del_1F	GCATGGGACTGAGTCTGGTC	
Amplification of 5' flank region	Del_2R	GAGTGTCTGAAGGTGCAGCTTCTCTTCCTCAGGGTCTCCG	The 5' end 20 nucleotides are complementary to P6R primer.
Amplification of 3' flank region	Del_3F	CGGGGATTATGCCTGGCTTTTGACTTTGGGATCTTGTGTTGA	The 5' end 20 nucleotides are complementary to P5F primer.
Amplification of 3' flank region and preparation of replacement construct 2	Del_4R	AGAAACGGAACTAGCGGCTC	
Amplification of *pyrG* gene	P5F	AAAGCCAGGCATAATCCCCG	
Amplification of *pyrG* gene	P6R	AGCTGCACCTTCAGACACTC	
Preparation of replacement construct 2	PY-F	ACAGCCGACTCAGGAGTTTG	
Preparation of replacement construct 1	YR-R	AACCATCACCGGTCTGAAGG	
Confirmation of *adh1* gene replacement	Check_1F	CTAGAAGATGCTACCCGCCG	Check_1F and Check_1R pair amplifies 5' flanking to 3' flanking region of *adh1* gene.
Confirmation of *adh1* gene replacement	Check_1R	CGCAACCTCCTTTCTTCCCT	
Confirmation of *adh1* gene replacement	Check_2F	GGTTACCGACGTAGCTTCCC	Check_2F and Check_2R pair amplifies inside *adh1* gene.
Confirmation of *adh1* gene replacement	Check_2R	GAGATGCAGTGGGCTCAAGT	
